# Genetic Adaptation and Acquisition of Macrolide Resistance in Haemophilus spp. during Persistent Respiratory Tract Colonization in Chronic Obstructive Pulmonary Disease (COPD) Patients Receiving Long-Term Azithromycin Treatment

**DOI:** 10.1128/spectrum.03860-22

**Published:** 2022-12-08

**Authors:** Anna Carrera-Salinas, Aida González-Díaz, Rachel L. Ehrlich, Dàmaris Berbel, Fe Tubau, Xavier Pomares, Junkal Garmendia, M. Ángeles Domínguez, Carmen Ardanuy, Daniel Huertas, Alicia Marín, Conchita Montón, Joshua Chang Mell, Salud Santos, Sara Marti

**Affiliations:** a Microbiology Department, Hospital Universitari de Bellvitge, IDIBELL-UB, Barcelona, Spain; b Research Network for Respiratory Diseases (CIBERES), ISCIII, Madrid, Spain; c Department of Microbiology and Immunology, Center for Genomic Sciences, Drexel University College of Medicine, Philadelphia, Pennsylvania, USA; d Department of Respiratory Medicine, Hospital de Sabadell, Hospital Universitari Parc Taulí, Institut d’Investigació i Innovació Parc Taulí I3PT, Universitat Autònoma de Barcelona, Sabadell, Spain; e Instituto de Agrobiotecnología, CSIC-Gobierno de Navarra, Mutilva, Spain; f Research Network for Infectious Diseases (CIBERINFEC), ISCIII, Madrid, Spain; g Department of Pathology and Experimental Therapeutics, School of Medicine, University of Barcelona, Barcelona, Spain; h Department of Respiratory Medicine, Hospital Residència Sant Camil, Consorci Sanitari Alt Penedès-Garraf, Barcelona, Spain; i Department of Respiratory Medicine, Hospital Universitari Germans Trias i Pujol, Barcelona, Spain; j Department of Respiratory Medicine, Hospital Universitari de Bellvitge, IDIBELL-UB, Barcelona, Spain; k Department of Medicine, School of Medicine, University of Barcelona, Barcelona, Spain; The University of Auckland

**Keywords:** *Haemophilus influenzae*, *Haemophilus parainfluenzae*, persistence, macrolide resistance, azithromycin, adaptation

## Abstract

Patients with chronic obstructive pulmonary disease (COPD) benefit from the immunomodulatory effect of azithromycin, but long-term administration may alter colonizing bacteria. Our goal was to identify changes in Haemophilus influenzae and Haemophilus parainfluenzae during azithromycin treatment. Fifteen patients were followed while receiving prolonged azithromycin treatment (Hospital Universitari de Bellvitge, Spain). Four patients (P02, P08, P11, and P13) were persistently colonized by H. influenzae for at least 3 months and two (P04 and P11) by *H. parainfluenzae*. Isolates from these patients (53 H. influenzae and 18 *H. parainfluenzae*) were included to identify, by whole-genome sequencing, antimicrobial resistance changes and genetic variation accumulated during persistent colonization. All persistent lineages isolated before treatment were azithromycin-susceptible but developed resistance within the first months, apart from those belonging to P02, who discontinued the treatment. H. influenzae isolates from P08-ST107 acquired mutations in 23S rRNA, and those from P11-ST2480 and P13-ST165 had changes in L4 and L22. In *H. parainfluenzae*, P04 persistent isolates acquired changes in *rlmC*, and P11 carried genes encoding MefE/MsrD efflux pumps in an integrative conjugative element, which was also identified in H. influenzae P11-ST147. Other genetic variation occurred in genes associated with cell wall and inorganic ion metabolism. Persistent H. influenzae strains all showed changes in *licA* and *hgpB* genes. Other genes (*lex1*, *lic3A*, *hgpC*, and *fadL*) had variation in multiple lineages. Furthermore, persistent strains showed loss, acquisition, or genetic changes in prophage-associated regions. Long-term azithromycin therapy results in macrolide resistance, as well as genetic changes that likely favor bacterial adaptation during persistent respiratory colonization.

**IMPORTANCE** The immunomodulatory properties of azithromycin reduce the frequency of exacerbations and improve the quality of life of COPD patients. However, long-term administration may alter the respiratory microbiota, such as Haemophilus influenzae, an opportunistic respiratory colonizing bacteria that play an important role in exacerbations. This study contributes to a better understanding of COPD progression by characterizing the clinical evolution of H. influenzae in a cohort of patients with prolonged azithromycin treatment. The emergence of macrolide resistance during the first months, combined with the role of Haemophilus parainfluenzae as a reservoir and source of resistance dissemination, is a cause for concern that may lead to therapeutic failure. Furthermore, genetic variations in cell wall and inorganic ion metabolism coding genes likely favor bacterial adaptation to host selective pressures. Therefore, the bacterial pathoadaptive evolution in these severe COPD patients raise our awareness of the possible spread of macrolide resistance and selection of host-adapted clones.

## INTRODUCTION

Chronic obstructive pulmonary disease (COPD) is a respiratory disorder characterized by airflow obstruction and inflammation, which results in chronically decreased lung function and respiratory failure. Although tobacco smoking is the main risk factor, other environmental and genetic factors increase the occurrence of COPD (1, 2). Acute exacerbations, characterized by increased airway inflammation and lower airway bacterial infection, represent an additional burden, requiring hospitalization, worsening comorbidities, and increasing mortality rates (3, 4).

The prophylactic use of azithromycin in COPD has been demonstrated to reduce the frequency and severity of exacerbations and improve the quality of life due to its immunomodulatory and anti-inflammatory properties (3–5). However, azithromycin is also a potent antibiotic that inhibits bacterial protein synthesis and interferes with the assembly of the 50S large ribosomal subunit (6). Its antimicrobial properties may affect the respiratory microbiota, including selecting for bacterial resistance to macrolides, which can arise due to mutations in several genes (the 23S rRNA gene near the encoded macrolide-binding region [A2058], several genes encoding rRNA methylases, and genes encoding the L4 and L22 ribosomal proteins) or by acquisition of efflux pumps encoded by the *mef*(A) and *msr*(D) genes (7–9).

Bacterial colonization is an important stimulus for inflammation and plays an important role in modulating exacerbations. Haemophilus influenzae is an opportunistic pathogen that normally colonizes the nasopharynx but is also the main etiologic agent in COPD exacerbations, particularly nontypeable strains (NTHi) (10). On the other hand, *H. parainfluenzae* is a frequent colonizer of the upper respiratory tract, although its role in COPD infection is unclear (11, 12). Different mechanisms in the bacteria, such as biofilm formation and intracellular survival, contribute to persistent colonization through evasion of the immune system and the action of antibiotics (13). In-host bacterial evolution during persistent COPD infections can occur through point mutations and DNA rearrangements, as well as through the acquisition or loss of mobile genetic elements, such as plasmids, bacteriophages, and pathogenicity islands that contain virulence factors (13, 14). Furthermore, Haemophilus spp. are naturally competent, and highly abundant DNA uptake signal sequences in their genomes (USSs: 5′-AAGTGCGGT-′3) promote exogenous DNA uptake and recombination among different strains (15).

Understanding the adaptive evolution of chronic bacterial pathogens to long-term drug selective pressure may improve our understanding of their role in COPD progression. Thus, the goals of this study were to characterize the H. influenzae and *H. parainfluenzae* population colonizing the respiratory tract in COPD patients that were treated with prolonged prophylactic azithromycin treatment to identify changes in azithromycin susceptibility and to detect genetic changes arising during persistent colonization.

## RESULTS

### Identification of respiratory bacterial isolates in COPD patients.

A total of 15 patients with severe COPD and frequent acute exacerbations were followed retrospectively before treatment and prospectively while receiving long-term azithromycin treatment (250 mg, 3 days/week) to characterize the opportunistic pathogens of their respiratory tract (Fig. S1 in the supplemental material). The most frequently isolated bacterial species in retrospective samples and those collected on the day of inclusion before starting the treatment (V1) were Moraxella catarrhalis (seven patients), followed by H. influenzae (six patients), and Pseudomonas aeruginosa (five patients).

On the other hand, cultured microbiota shifted after the onset of azithromycin treatment (V2, V3, and acute exacerbations): *H. parainfluenzae* was detected at least once in 10 of the 15 patients, followed by H. influenzae and P. aeruginosa (isolated from seven patients, each) and Stenotrophomonas maltophilia (six patients).

### Characterization of persistent colonization of the respiratory tract by H. influenzae and *H. parainfluenzae*.

Persistent lineages were defined as those belonging to the same molecular type (differing by no more than one multilocus sequence typing [MLST] locus or differing by three or fewer pulse-field gel electrophoresis [PFGE] bands in H. influenzae and *H. parainfluenzae*, respectively) and were isolated in the same patient for periods of more than 3 months. Concurrent colonization by nonpersisting strains was also observed; however, despite characterizing 10 colonies per episode, it is possible that persistence rates were higher due to polyclonal infections.

**FIG 1:**
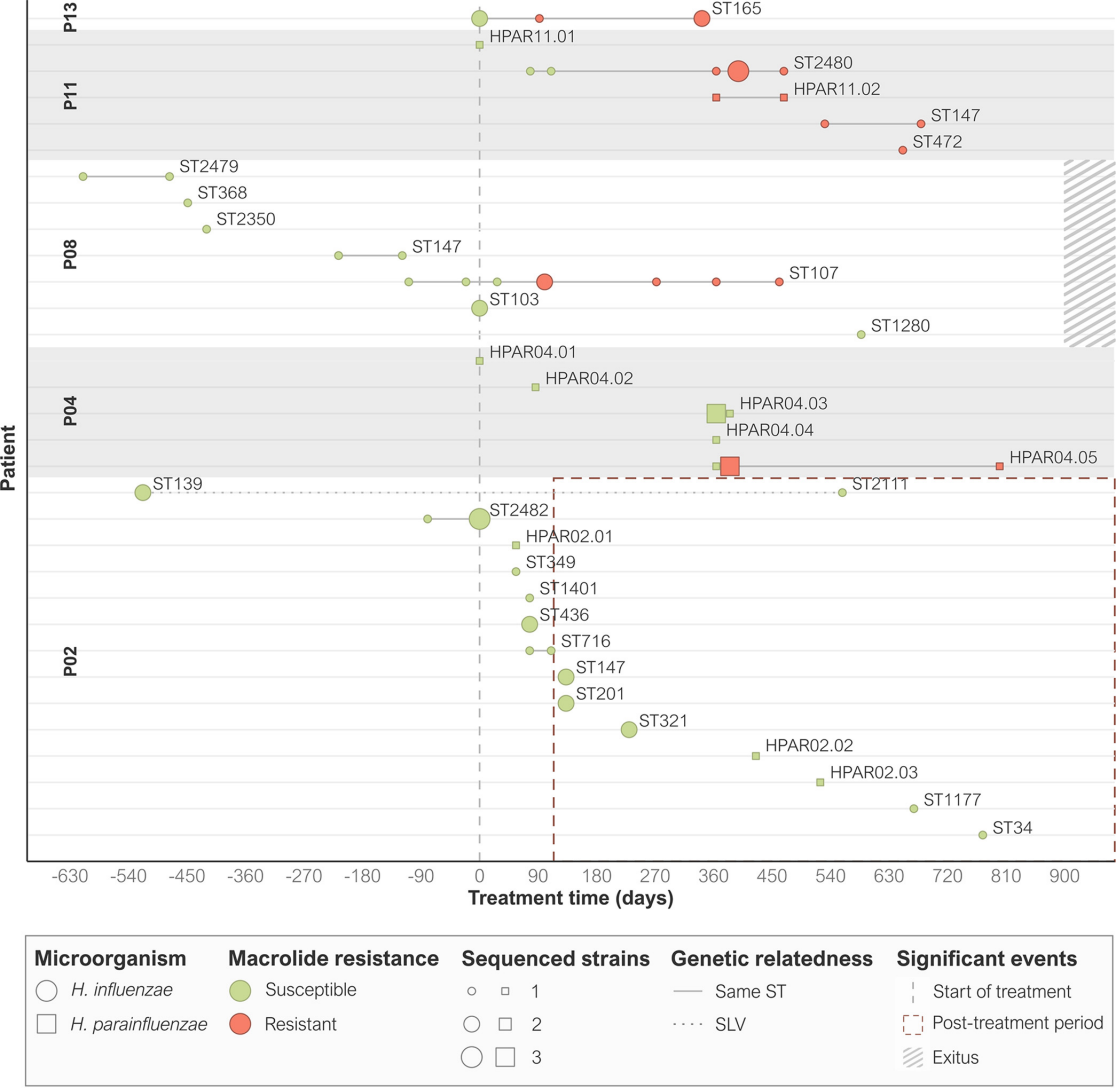
Timeline illustrating H. influenzae and *H. parainfluenzae* strains isolated from respiratory samples in COPD patients with long-term azithromycin therapy. Persistence was defined as the isolation of the same clone in a patient for more than 3 months based on the ST and PFGE pattern in H. influenzae and *H. parainfluenzae*, respectively. Persistence is depicted as a gray line connecting the isolates. ST, sequence type; SLV, single-locus variant.

Five of 15 patients had persistent H. influenzae or *H. parainfluenzae* colonization, and therefore, isolates from these patients were included for whole-genome sequencing (WGS) characterization (Fig. 1): four patients (P02, P08, P11, and P13) were persistently colonized by H. influenzae (Data set S1A), and two (P04 and P11) were persistently colonized by *H. parainfluenzae* (Data set S1B). Despite persistent H. influenzae and *H. parainfluenzae* colonization, control of acute exacerbations in these patients at 1-year follow-up (1.25 ± 0.96) was comparable to those without persistent colonization (2.30 ± 1.42) (*P* = 0.2030).

A brief description of persistent colonization in the five patients is described below. Although patient P02 withdrew from azithromycin treatment early in the study due to adverse effects, the colonization pattern of H. influenzae isolates was examined, identifying 11 distinct PFGE patterns but only 1 possible case of persistence, in which 2 closely related strains were isolated more than 3 years apart (sequence type 139 [ST139] and single-locus variant [SLV] ST2111).

P04 was colonized by five different *H. parainfluenzae* clones. One of these, belonging to PFGE-type HPAR04.05, was isolated during an acute exacerbation 1 year after treatment began and continued to persistently colonize the patient for at least 14 months after its initial detection.

Before treatment, P08 had two cases of persistent colonization by ST147 and ST2479 clones, but these were not further isolated during azithromycin treatment. ST107 clone was first identified 3 months before treatment and was repeatedly isolated during treatment, both in stable phases and acute exacerbations, for at least 570 days.

P11 was persistently colonized by two H. influenzae lineages (ST2480 and ST147) and one *H. parainfluenzae* (HPAR11.02). ST2480 was identified 2 months after beginning treatment and was isolated for over a year at stable and acute exacerbation episodes; ST147 was detected after 17 months of treatment and persisted for at least 5 months; and HPAR11.02 was detected 1 year after starting treatment and lasted at least 104 days.

Finally, P13 was persistently colonized by a single H. influenzae lineage belonging to ST165, which was isolated during the first year of treatment at the stable-phase control visits.

### Macrolide resistance development during long-term azithromycin therapy.

All strains isolated before treatment were susceptible to azithromycin but after starting the therapy, newly isolated clones had developed resistance within the first months of treatment (Table 1), except for those from patient P02, who discontinued the treatment (Fig. 1).

**TABLE 1:** Azithromycin resistance and macrolide resistance determinants in persistent H. influenzae and *H. parainfluenzae* strains^*a*^

Patient	Microorganism	PFGE pattern	ST	Treatment time (days)	Azithromycin resistance			Macrolide resistance determinants	
					S/R	Disk diffusion (mm)	Microdilution (mg/L)	Genetic determinant	Alteration
P02	H. influenzae	HINF02.05	ST139	−518	S	27	2		
		HINF02.05	ST2111^*b*^	558	S	24	2		
P04	*H. parainfluenzae*	HPAR04.05		364	S	21	4		
		HPAR04.05		385	R	11	16		
		HPAR04.05		800	R	0	32	L1	D85G
								S1	T327A
								RlmC	F193L
P08	H. influenzae	HINF08.04	ST2479	−610; −477	S	23	2		
	H. influenzae	HINF08.06	ST147	−217; −119	S	19	2		
	H. influenzae	HINF08.01	ST107	−109; −21	S	24	4		
				27	S	19	4	*acrR*	Q11STOP
				100	R	0	128	*acrR*	Q11STOP
								23S rRNA	A2058G (50% copies)
				272; 364; 461	R	0	256	*acrR*	Q11STOP
								23S rRNA	A2058G (100% copies)
P11	H. influenzae	HINF11.01	ST2480	78; 110	S	19	4		
				364; 398; 468	R	0	256	L22	78-GPSMKRVMPRAK-79
	*H. parainfluenzae*	HPAR11.02		364; 468	R	0	8	MsrD/MefE	
	H. influenzae	HINF11.02	ST147	531; 679	R	0	256	MsrD/MefE	
P13	H. influenzae	HINF13.01	ST165	0	S	18	4		
				92	R	0	128	L22	K90E
				342	R	0	128	L22	K90E
								L4	G65D

a Negative values for treatment time indicate days before starting azithromycin treatment. ST, sequence type; S, susceptible; R, resistant.

b SLV, single-locus variant.

In the persistent *H. parainfluenzae* HPAR04.05 isolates (P04), the initial strain was susceptible to azithromycin (MIC = 4 mg/L). The next isolate was resistant to azithromycin (MIC = 16 mg/L), and the last isolate had even higher resistance levels (MIC = 32 mg/L), along with predicted resistance mutations in genes encoding the ribosomal proteins L1 (D85G), S1 (T327A) and 23S rRNA (uracil(747)-C(5))-methyltransferase (RlmC) (F193L).

Two cases of persistence by H. influenzae ST147 and ST2479 in patient P08 were susceptible to azithromycin, since they were isolated before treatment and thus not under macrolide pressure. However, serial isolates of ST107, which was first isolated before treatment, showed serially increasing resistance, strongly suggesting adaptive evolution of a single persistent clone. The first strain isolated after starting treatment was susceptible, despite acquiring a premature stop codon in *acrR*, encoding the AcrAB-TolC efflux pump repressor. After 100 days of treatment, the ST107 clone was highly resistant (MIC = 128 mg/L), accompanied by three of the six copies of the 23S rRNA with an A2058G mutation, in addition to the premature stop in *acrR*. Later isolates of ST107 had even higher resistance (MIC = 256 mg/L), and all copies of 23S rRNA had acquired the A2058G mutation.

In patient P11, H. influenzae isolates of ST2480 were initially susceptible but then developed azithromycin resistance (MIC = 256 mg/L) within 1 year of treatment after an insertion in the gene encoding ribosomal protein L22. In contrast, a persistent azithromycin-resistant *H. parainfluenzae* HPAR11.02 clone (MIC = 8 mg/L) was isolated after a year of treatment, due to acquisition of the macrolide efflux genetic assembly (MEGA) element with the *msr*(D) and *mef*(E) genes. The MEGA element was also found in the subsequent persistent azithromycin-resistant H. influenzae ST147 clones also isolated in patient P11 (MIC = 256 mg/L). It should be noted that ST147 clones isolated in other patients (P02 and P08) did not have the MEGA element. This suggests horizontal gene transfer of the MEGA element from an unknown donor into *H. parainfluenzae*, followed by subsequent transfer to ST147.

In the persistent clone ST165 from P13, the initial susceptible strain developed azithromycin resistance after 3 months of treatment (MIC = 128 mg/L), also due to substitutions in the L22 ribosomal protein (K90E) and the L4 ribosomal protein (G65D).

In addition to azithromycin, patients also received amoxicillin-clavulanic acid, levofloxacin, ciprofloxacin, and second-(cefuroxime) and third-generation cephalosporins (ceftizidime, cefuroxime, and cefditoren) to treat infections that developed during acute exacerbations. These antibiotic treatments were not linked to the development of antimicrobial resistance (Data set S1A and S1B).

### Phylogeny, population structure, and horizontal gene transfer.

**FIG 2:**
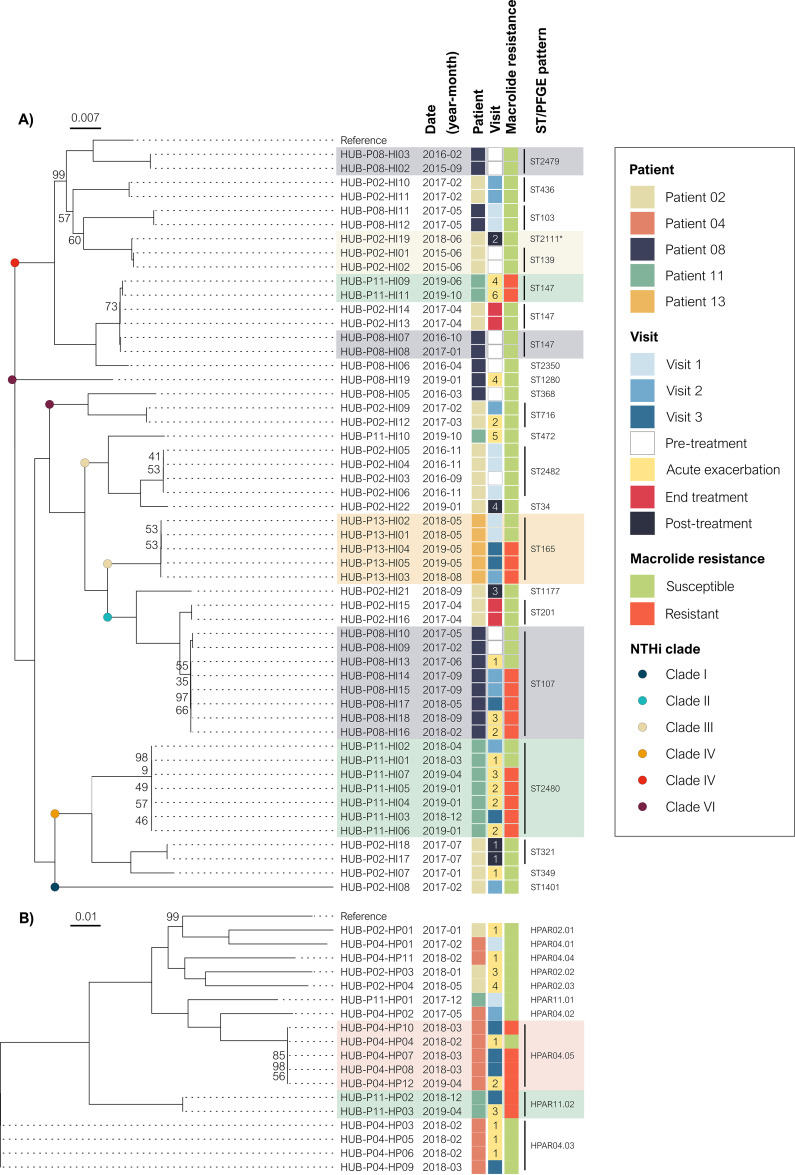
Phylogenetic tree of Haemophilus spp. isolated from COPD patients during long-term azithromycin treatment. (A) H. influenzae phylogenetic tree. NTHi were classified into six clades (I to VI), as previously described (48, 49). Strain Hi375 (CP009610) was used as the reference. (B) *H. parainfluenzae* phylogenetic tree. Strain T3T1 (NC_015964) was used as the reference. Persistence was defined as the isolation of the same lineage in a patient for more than 3 months based on the ST in H. influenzae isolates or the PFGE pattern in *H. parainfluenzae* isolates. Shadowed areas highlight persistent lineages. The numbers in the acute exacerbation and posttreatment boxes are used to distinguish between episodes. Bootstrap values other than 100 are given at branch nodes. *SLV, single-locus variant.

All the H. influenzae (*n* = 53) and *H. parainfluenzae* (*n* = 18) isolates sequenced were noncapsulated. Phylogenetic analysis revealed a diverse population in both species, H. influenzae (Fig. 2A) and *H. parainfluenzae* (Fig. 2B). *H. parainfluenzae* strains showed greater genetic heterogeneity and phylogenetic distance than H. influenzae strains. However, strains belonging to each persistent lineage formed well-defined clades with high genetic similarity. Classification of the NTHi strains based on the presence or absence of 17 accessory genes revealed that all were distributed among the 6 major clades in the phylogenetic tree with no evidence of large clonal expansions (Fig. 2A).

**FIG 3:**
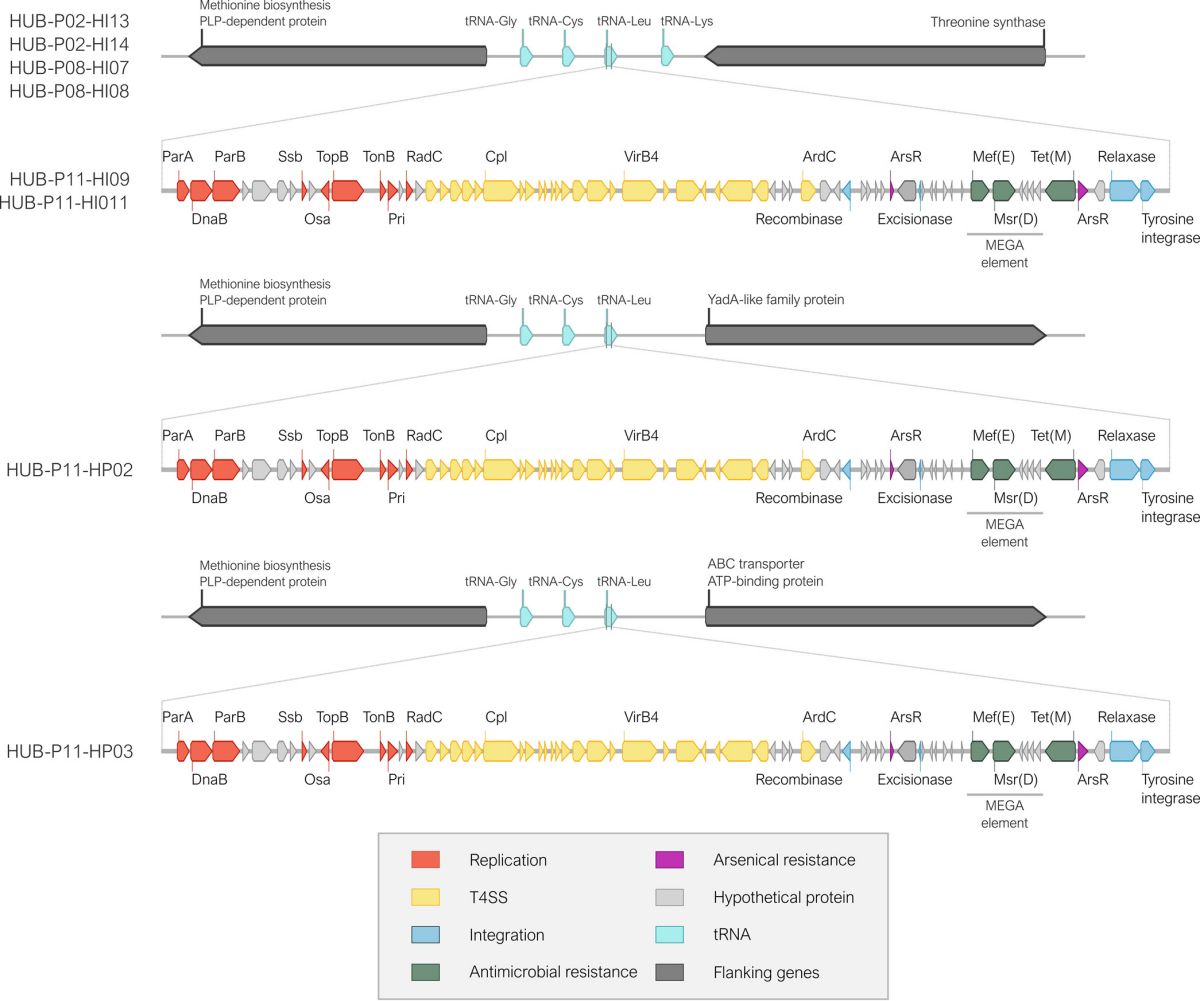
Schematic representation of ICE*HpaHUB5* structure containing *tet*(M)-MEGA element observed in H. influenzae ST147 (HUB-P11-HI09/011) and *H. parainfluenzae* HPAR11.02 (HUB-P11-HP02/03) clones from P11. The detected MEGA element was the same as that found in the *H. parainfluenzae* strain AE-2096513 (KJ545575).

MLST analysis also highlights the high heterogeneity in the NTHi population from these five patients, detecting 21 different STs among the 53 strains. Only ST147 was found in different patients (P02, P08, and P11), which was also associated with two cases of persistence (in P08 and P11). The comparison of the ST147 strains isolated from these patients revealed that those from P11 had a 60-kb insertion in a gene for tRNA-Leu, between the genes encoding the methionine biosynthesis PLP-dependent protein and threonine synthase, which was not found in the ST147 strains from P02 and P08. This insertion corresponded to an integrative conjugative element (ICE) called ICE*HpaHUB5*, which contained 70 genes, including genes involved in replication, type IV secretion system, and integration. This ICE also contained the MEGA element, which carries *msr*(D) and *mef*(E) genes adjacent to *tet*(M) and was inserted into the *arsR* gene encoding an arsenical resistance repressor (Fig. 3). Interestingly, this 60 kb region was also detected in *H. parainfluenzae* HPAR11.02 isolates (HUB-P11-HP02 and HUB-P11-HP03) from P11, inserted within distinct tRNA-Leu genes and showing a high percentage of identity (100% and 99.997%, respectively) with the ICE found in the H. influenzae ST147 strains from P11, suggesting the probable and recent horizontal transference between the strains isolated from P11. *H. parainfluenzae* HPAR11.02 isolates carrying the ICE were identified before the isolation of H. influenzae ST147 strains from P11, suggesting that the 60-kb region could have been transferred from *H. parainfluenzae* to H. influenzae isolates.

### Genetic changes observed during persistent colonization.

**FIG 4:**
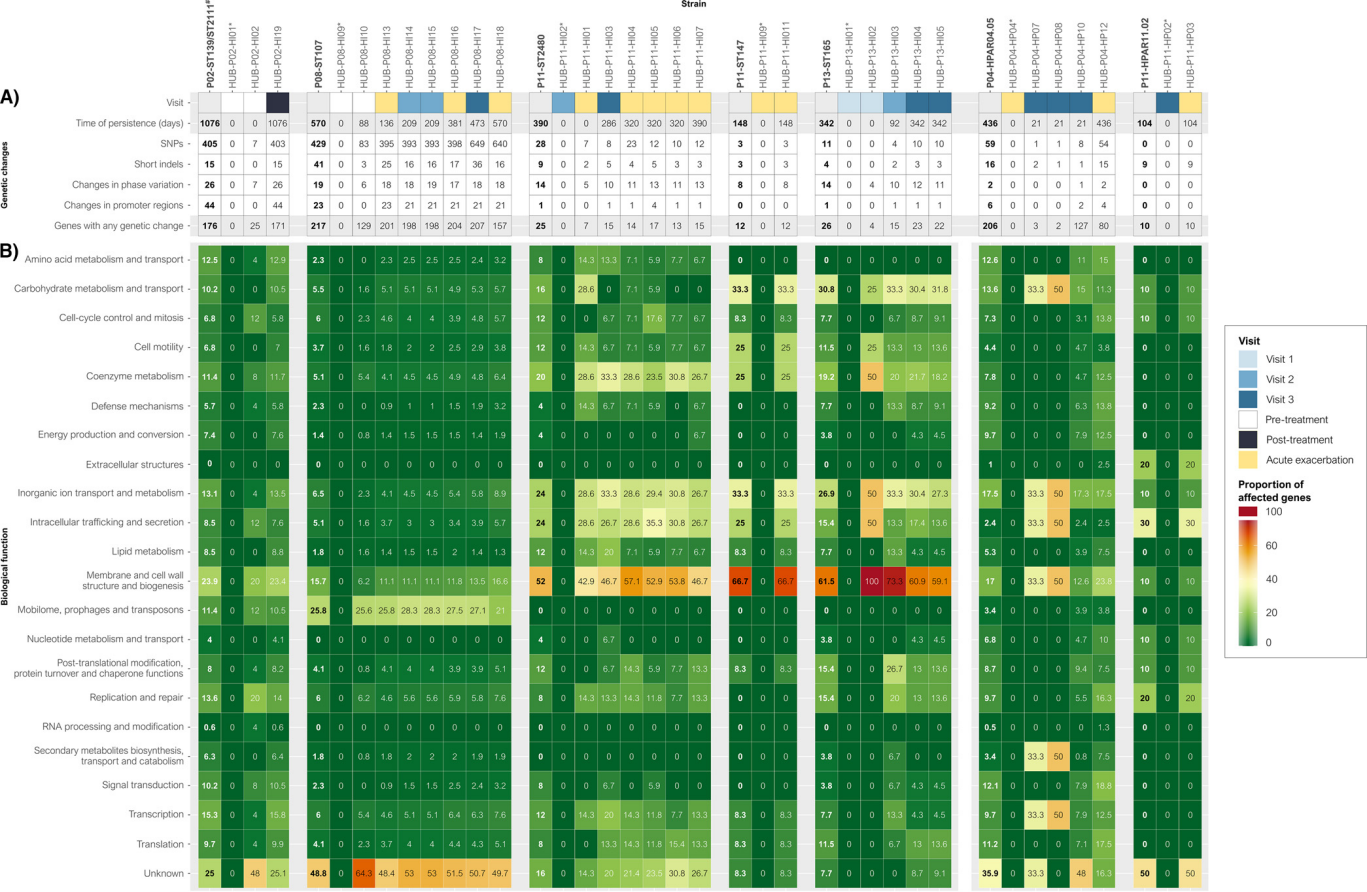
Genetic changes and biological functions of the affected genes in each persistent case. (A) Number of genetic changes, including nonsynonymous SNPs, short indels (≤10 bp), and changes in phase variation and promoter regions, taking place during persistent colonization and the number of genes that presented any of these changes. (B) Biological functions of the genes affected in each persistent case. The numbers inside the boxes represent the percentage (%) of genes with any genetic change that had each biological function. Each gene may be annotated with more than one biological function, so that the sum of all the percentages may be >100%. In the first column of each persistence case, the overall proportions of genes that were altered are highlighted in bold. *The first persistent strain isolated over time with a closed genome was used as the reference in each persistence case.

Genome-wide changes arising in serially collected persistent isolates were examined to determine additional changes taking place in Haemophilus spp. during prolonged azithromycin treatment (Fig. 4A and Data set S2), in addition to those potentially associated with macrolide resistance. ST147 and ST2479 persistent isolates from P08 were excluded from this analysis since they were only isolated before treatment.

Most genomic variation among closely related persistent strains occurred in genes encoding proteins with unknown functions, followed by genes associated with membrane and cell wall structure and inorganic ion transport and metabolism. Figure 4B depicts the biological functions of the genes undergoing genetic variation in each persistence case, and Data set S3 contains the proportion of genes belonging to each biological function in reference genomes.

Among all the genetic changes, 16 genes stood out as having acquired genetic alterations across serially collected isolates over time in H. influenzae (Table S1), while in *H. parainfluenzae* all the changes were unique to each case of persistence. In all cases of persistence by H. influenzae, mutations were observed for *hgpB* and *licA* genes, which encode hemoglobin-haptoglobin binding protein B and phosphorylcholine kinase LicA, respectively. These changes were mostly associated with phase variation at simple sequence repeats within the open-reading frames (*hgpB*, 5′-CCAA-3′; *licA*, 5′-CAAT-3′), which frameshifts protein translation and introduces premature stop codons. Nonsynonymous single-nucleotide polymorphisms (SNPs) were also found in the *hgpB* gene of the P02-ST139/ST2111 and P08-ST107 clones and in the *licA* gene of P08-ST107 clone. Other genes that had allelic changes in independent lineages and patients were *hgpC*, coding for hemoglobin-haptoglobin binding protein C; *fadL* (*ompP1*), coding for an outer membrane transporter; *lic3A*, coding for CMP-Neu5Ac-lipooligosaccharide α-2,3-sialyltransferase; *lex1*, coding for lipooligosaccharide biosynthesis protein Lex-1; and genes coding for glycosyltransferase family 2 and 8 proteins (Fig. S2). Most genetic changes in these genes were related to phase variation at simple-sequence repeats; however, changes to *fadL* were associated with distinct single-base indels that caused frameshifting and premature stop codons at different positions without exhibiting any truncation pattern throughout persistence (Fig. S2D).

### Gain, loss, and evolution of genome-integrated prophages during persistent infection.

**FIG 5:**
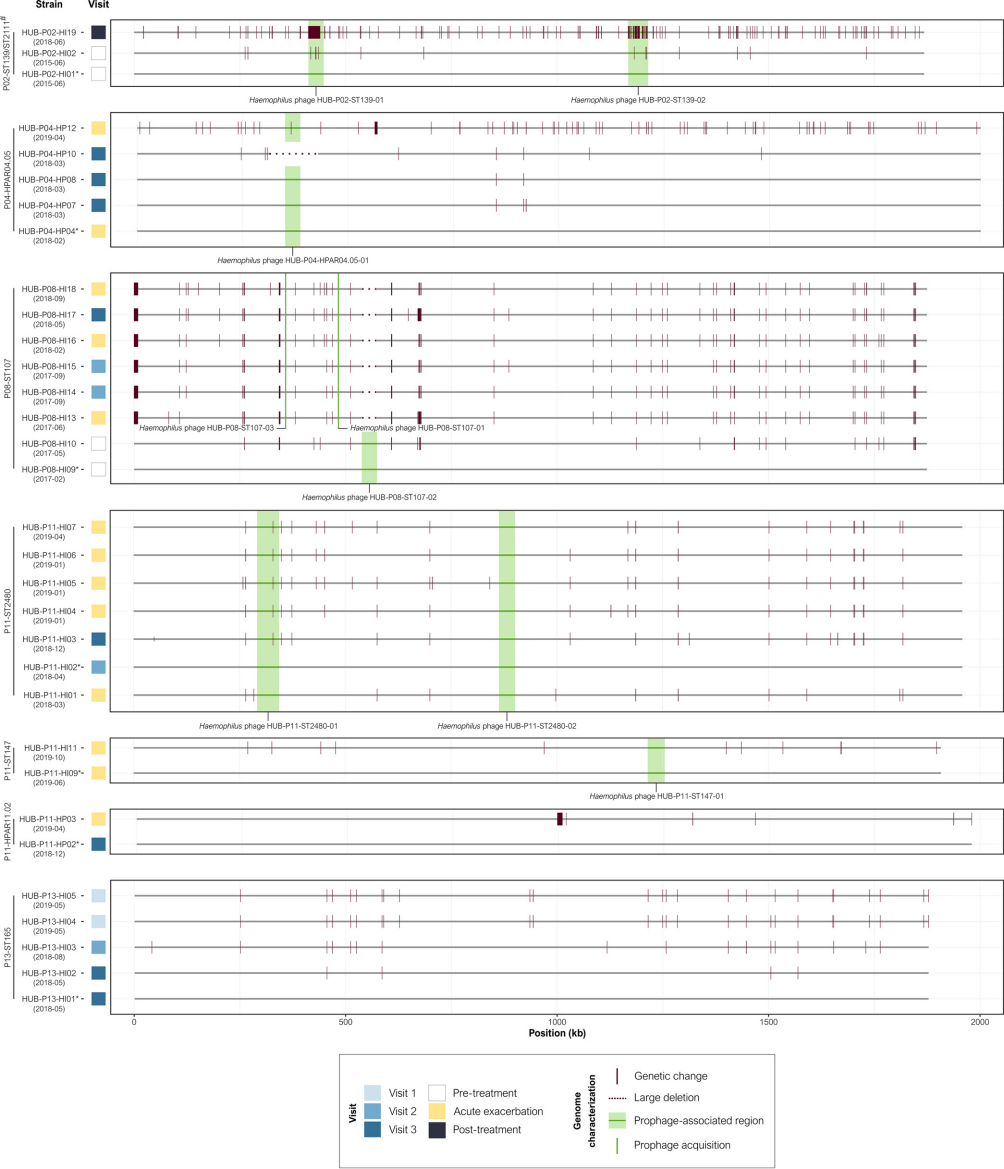
Alignment of whole-genome sequences of persistent Haemophilus spp. isolates. Assemblies from each persistence episode were aligned using the first isolate from each series as the complete genome reference (*) for the lineage. Prophage-associated regions were identified by Phaster. Acquisition date is indicated in brackets (year-month). ^#^SLV, single-locus variant.

Large insertion and deletion polymorphisms were discovered in some cases of persistence (Fig. 5). An in-depth examination of these regions revealed gain and loss of genomic prophages, as well as, in some cases, accumulated mutations within the prophages over time.

H. influenzae strains associated with ST139/ST2111 from patient P02 had two genome-integrated prophages (Haemophilus phage HUB-P02-ST139-01 and Haemophilus phage HUB-P02-ST139-02) (Fig. S3A and B), which had accumulated most of the SNPs (261/405) acquired over time. In the last isolated strain (HUB-P02-HI19), Haemophilus phage HUB-P02-ST139-02 showed 90.4% of identity to the reference strain (HUB-P02-HI01). The location of this prophage within the genome is the same over time, suggesting the probable recombination with another related prophage from an unknown donor.

In patient P04, persistent isolates of *H. parainfluenzae* HPAR04.05 had *the* Haemophilus phage HUB-P04-HPAR04.05-01 prophage (Fig. S3C), except for strain HUB-P04-HP10, which had lost 115,160 bp corresponding to the prophage and its surrounding regions (37,453 bp downstream and 41,640 bp upstream) (Data set S4).

Acquisition and loss of genome-integrated prophages over time was detected in the patient P08 persistent H. influenzae ST107 isolates. However, the prophages detected in these strains remained unchanged over time (Fig. S3D and F), similar to the three prophages found in the P11 persistent lineages, which showed high stability throughout the serial bacterial isolates (Fig. S3G to I).

The USS motif is found every ~1.2 kb of a Haemophilus genome, and these accumulate by mutation and biased uptake over long evolutionary timescales (16, 17); therefore, the frequency of USS within prophage insertions can be an indicator of how long a given phage has been resident in Haemophilus species. Similarly, although horizontally transferred phage from other species may have various guanine-cytosine (GC) content from the new host, the GC content evolves toward a host-adapted GC content over time. Thus, deviations in prophage USS density or GC content are indicators of how recently phage-host coevolution began (Table S2). On average, Haemophilus prophage sequences in the persistent isolates had a density of 0.41 USS/Kb (SD = 0.13), significantly lower than the whole-genome density, which had 0.78 USS/Kb (SD = 0.03) (*P* < 0.0001), and in all cases, USS density was lower in individual prophages compared to the genome average, indicating their relatively recent acquisition of Haemophilus. Different prophage sequences ranged from 0.26 to 0.69 USS/kb, suggesting some (e.g., Haemophilus phage HUB-P02-ST139-02) have been resident in Haemophilus much longer than others (e.g., Haemophilus phage HUB-P11-ST2480-02). Similarly, average GC content across prophages was 41.0% (SD = 2.03) compared to 38.3% (SD = 0.44) in whole-genome sequences (*P* < 0.0001), suggesting their origin in other bacterial groups. In all prophages and genomes, GC content remained stable over time, as expected, although extensive variation added to Haemophilus phage HUB-P02-ST139-01 decreased GC content from 39.3% to 38.3%, comparable to the genome where it was integrated (38.1%).

**FIG 6:**
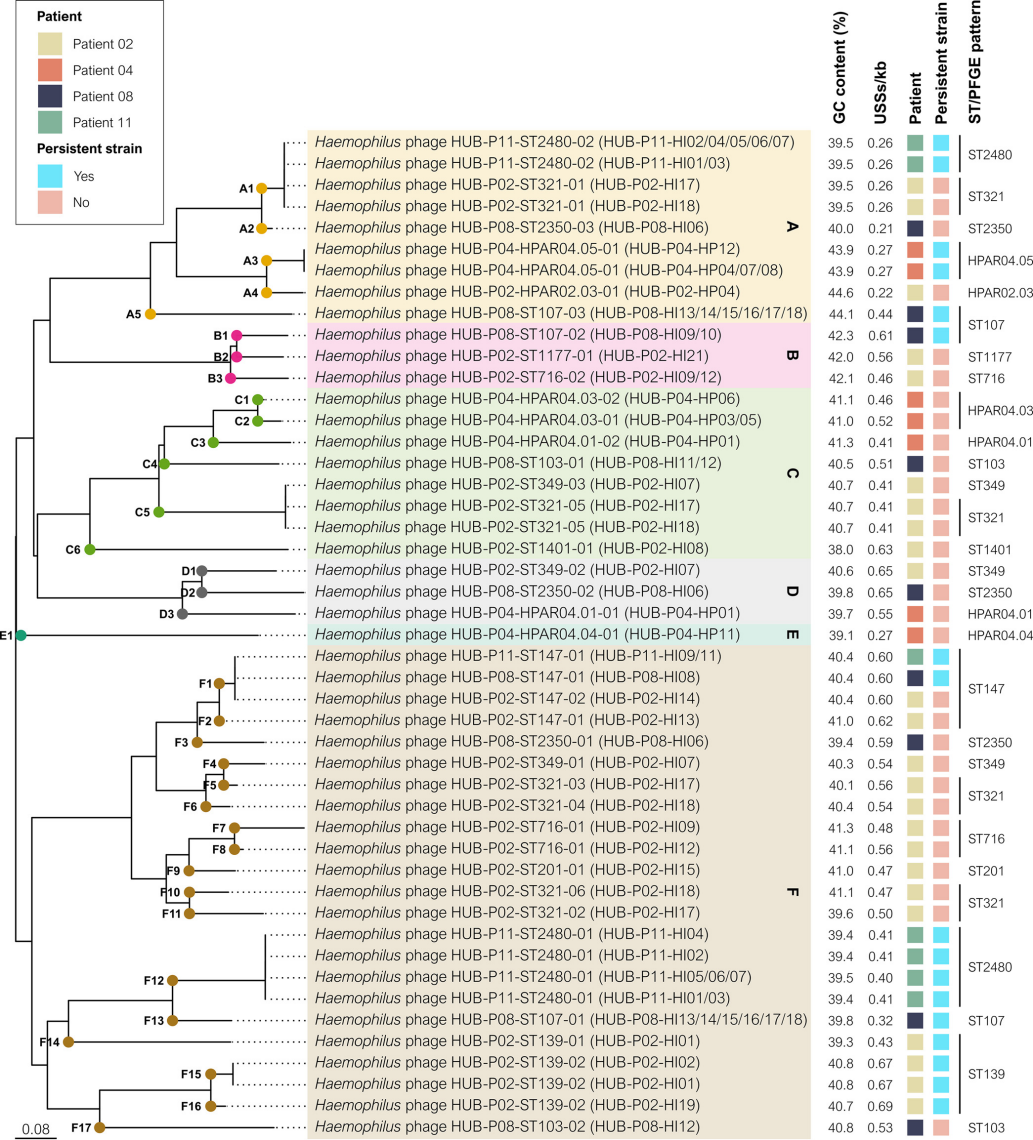
Phylogenetic tree of genome-integrated prophages detected in Haemophilus spp. strains. VICTOR software was used to calculate genome-BLAST distance phylogeny (GBDP) using D0 formula to estimate phylogeny from nucleotide sequences. The name of the prophages is colored according to the genus clusters (A to F), and the dots arranged on the branches of the tree represent the different species clusters identified by VICTOR software. GC, guanine-cytosine; USS, DNA uptake signal sequences.

Prophages identified in all the H. influenzae and *H. parainfluenzae* strains (*n* = 72) from the patients included in this study were analyzed phylogenetically (Fig. 6): all of them belonged to the same family cluster and were classified into 6 genera (A to F) and 35 species clusters. There were no significant differences in GC content between genus clusters, although prophages from genus cluster A had fewer USS per kilobase (0.27 USS/kb, SD = 0.07) than prophages from other genus clusters, suggesting that it only more recently began infecting Haemophilus species. Moreover, in some cases, prophages identified in different patients and/or different clones belonged to the same species cluster, suggesting a possible transmission of prophages between microorganisms present in the same patient or between different strains: both Haemophilus phage HUB-P02-ST321-01 and Haemophilus phage HUB-P11-ST2480-02 belonged to species cluster A1, despite coming from different STs isolated from different patients; and Haemophilus phage HUB-P02-ST321-05 and Haemophilus phage HUB-P02-ST349-03, identified in different STs from the same patient, belonged to species cluster C5. Furthermore, the ST147 strains from P02, P08, and P11 carried a prophage belonging to the same species cluster F1, and the closely related F2, suggesting that this prophage may have higher specificity for ST147 clones.

## DISCUSSION

Prolonged azithromycin treatment improves the quality of life in patients with severe COPD and recurrent exacerbations by modulating the immune system and decreasing inflammation in the respiratory tract (5, 6). However, our results indicate that long-term use of this antibiotic has major effects on the microbiota and favors the development of macrolide resistance through enhanced selection of resistant bacteria (18, 19).

An in-depth analysis of respiratory tract samples from COPD patients identified differences in lung colonization by bacterial pathogens after long-term macrolide treatment. Before therapy, M. catarrhalis and H. influenzae were the most frequently isolated species during both stable phases and exacerbations, as previously reported (20). However, once azithromycin treatment was initiated, *H. parainfluenzae*, H. influenzae, P. aeruginosa, and S. maltophilia became the most frequently isolated bacteria. In particular, we noted increased prevalence of *H. parainfluenzae* during treatment. This might be because *H. parainfluenzae* is considered nonpathogenic and could be undersampled during routine clinical microbiology practice. Some studies support the role of *H. parainfluenzae* in respiratory tract colonization in both healthy subjects and during respiratory disease (11, 12, 21, 22). However, unlike H. influenzae colonization, *H. parainfluenzae* does not produce an increased inflammatory response (23), suggesting that the importance of this microorganism may be related to its capacity for antimicrobial resistance acquisition allowing it to act as a reservoir for resistance genes (24–26) that, under azithromycin pressure, may spread to other related bacteria sharing the same ecological niche.

Long-term azithromycin therapy is associated with the development of macrolide resistance (18). A subtherapeutic but continuous dose is sufficient to induce macrolide resistance in both H. influenzae and *H. parainfluenzae* strains. Many of the genetic changes seen in persisting strains are consistent with changes to ribosomes that block macrolide binding. The A2058G mutation in the domain V of the 23S rRNA (7) was observed to arise in some persistent H. influenzae strains, and this change accumulated in the six different copies of the gene over long-term antibiotic therapy, resulting in multiple stepwise increases in macrolide resistance levels. Other genetic alterations linked to the acquisition of azithromycin resistance in persistently infecting H. influenzae strains were changes in the ribosomal proteins L4 and L22, which have been described previously (6, 7). Alterations in genes encoding ribosome-related proteins, including S1, L1, and RlmC, were also observed in some *H. parainfluenzae* macrolide-resistant strains. S1 is involved in translation, transcription, and RNA stability (27), whereas L1 is a component of the ribosomal 50S subunit that binds to 23S rRNA and participates in translation (28). RlmC is a methyltransferase that methylates U747 of 23S rRNA, promoting RlmA methylation of G748 and facilitating the binding of macrolides (29). Thus, changes in RlmC could indirectly affect macrolide binding to the ribosome, as previously suggested by Shoji et al. (30), who observed that disrupted RlmCD mutants had increased resistance to telithromycin in S. pneumoniae. However, additional research is required to confirm the role of S1, L1, and the specific RlmC mutation observed here (F193L) in mediating macrolide resistance. These results indicate that the microbiota in patients undergoing prolonged use of azithromycin should be monitored, since the treatment promotes the development and acquisition of resistance by respiratory tract colonizing pathogens. Macrolides are commonly used to treat respiratory infections, and the presence of colonizing bacteria with acquired and transmissible resistance mechanisms may complicate their treatment.

Genetic changes were also seen in persistent Haemophilus species that could confer macrolide resistance via efflux pumps. The 23S rRNA gene mutations were observed in strains that had previously acquired a premature stop codon in AcrR (Q11), a negative regulator that results in the overexpression of the AcrAB efflux pump. The change in AcrR produced no change in azithromycin susceptibility, indicating no direct association with resistance, although this change could enable subsequent mutations in other genes to provide resistance, as previously reported for *acrB* (31). Previous studies have described transferrable transposons in Streptococcus spp. and *H. parainfluenzae* that carry *tet*(M) and the MEGA element (8, 9), which encodes the Mef(E) and Msr(D) efflux pumps that can confer macrolide resistance. In this study, an integrative conjugative element carrying the *tet*(M)-MEGA element was found to arise in *H. parainfluenzae* strains and then later H. influenzae strains in the same patient. This pattern suggests the possibility of horizontal transmission between species sharing the same ecological niche within the patient, further supporting the role of *H. parainfluenzae* as a reservoir for resistance genes (24, 32).

During persistent colonization, genetic differences that arose were also usually seen in successive serial isolates, suggesting selective sweeps and bacterial adaptation. Most of the changes affecting genes of known function encode proteins involved in the structure of the bacterial cell surface. In fact, most of H. influenzae persistence cases showed alterations in *licA*, *lex1*, and *lic3A*. These changes likely reflect host immune pressures selecting for Haemophilus strains to alter their surface antigens or evade the effects of complement-mediated killing. The *licA* gene encodes a phosphorylcholine kinase, which adds phosphorylcholine to lipooligosaccharide (LOS) and interacts with C-reactive protein, activating the complement system. Phase variation of *licA* causes changes in its expression and, as a result, the amount of phosphorylcholine that coats the bacterial surface, which may interfere with bactericidal antibody access to the surface and affect strain virulence and sensitivity to serum bactericidal activity (33, 34). On the other hand, Lex1 is involved in LOS synthesis and *lic3A*, which encodes the α-2,3-sialyltransferase responsible for the addition of *N*-acetylneuraminic acid (Neu5Ac) to the LOS, may have phase variation changes, affecting epithelial cell adhesion, invasion, and serum resistance (35, 36).

Genes involved in inorganic ion metabolism and transport also had a high number of changes across persistently infecting isolates, and some genes coding for TonB-dependent receptors stand out, particularly *hgpB* and *hgpC*, which are involved in hemoglobin and hemoglobin-haptoglobin uptake to supply the requirement of H. influenzae for heme (37). Another gene that showed recurrent alterations was *fadL*, as observed by Moleres et al. (38), who described truncated variants, albeit in different positions than those in our study, which could affect the interaction with host cells and increase resistance to the bactericidal effects of long-chain fatty acids found in high abundance in the lungs of COPD patients. Other studies on the adaptation of H. influenzae to disease have also found changes in these genes (38–40), suggesting that rapid genetic change at these genes could provide a selective advantage that favors bacterial survival and colonization over long periods of time.

Most of the observed genetic changes were related to phase variation, causing frameshifting and premature stop codons, although other open-reading frames compatible with protein translation could appear. Therefore, it would be possible that some of these changes had no impact on protein function, and further research would be required to fully understand the impact of these genetic changes and their possible implication on the reversible switch in gene expression.

Genome-integrated prophages within H. influenzae and *H. parainfluenzae* genomes were common, and their acquisition and loss were frequently observed in serial isolates. Although phage can debilitate a bacterial population in the lytic phase, lysogenic prophages are potent vehicles of horizontal gene transfer, which may improve the ecological fitness of bacteria in certain conditions and persist in a latent state while coevolving with the bacterial chromosome (41). Some prophages showed high genetic changes, which could be due to recombination or selective pressures exerted by the bacteria themselves and can direct the prophage adaptation to the genomes where they have been integrated. These changes could potentially be used to track phage-host coevolution. Szafrański et al. (42) indicated that USSs have a higher density of core genes than accessory genes. Therefore, prophages in the lysogenic phase would accumulate more USSs as they evolved with the host, while also adapting the GC content to that of the genome into which they had been integrated (17, 42). Our prophages showed a wide range of USS densities, indicating that some of them have recently been acquired by H. influenzae and others have been within the species for a long time. However, additional research involving more prophages and also prophages identified in other bacteria is required.

In conclusion, the antibiotic pressure exerted by prolonged azithromycin treatment in COPD patients, even at low doses, leads to the development of macrolide resistance through the accumulation of mutations in 23S rRNA, mutations in ribosomal proteins, or the acquisition of efflux pumps through transmission of mobile genetic elements. Furthermore, during persistent colonization of the respiratory tract, H. influenzae and *H. parainfluenzae* acquire prophages and genetic changes, particularly in genes associated with membrane and cell wall, as well as inorganic ion metabolism and transport proteins, which likely improves their adaptation to host selective pressures and allows bacterial survival for long periods of time. To prevent the spread of resistant and highly host-adapted clones, further monitoring of bacterial pathoadaptive evolution is required.

## MATERIALS AND METHODS

### Study design and bacterial strains.

This was a laboratory-based study that included clinical respiratory isolates from a cohort of patients with severe COPD and frequent exacerbations (≥4 acute exacerbations, or ≥3 acute exacerbations with at least 1 hospitalization in the year before inclusion) who had initiated long-term oral azithromycin treatment (250 mg, 3 days/week) at the Hospital Universitari de Bellvitge (Spain). Patients were followed for at least 1 year, with sputum samples collected just prior to the start of azithromycin therapy (V1), 3 months after starting therapy (V2), 12 months after starting therapy (V3), and during any acute exacerbation episodes. Other respiratory bacterial isolates recovered during routine clinical microbiology laboratory work before initiating treatment were also included.

All the sputum samples were cultured on blood, chocolate, and MacConkey agar, and individual colonies were isolated and identified by mass spectrometry using matrix-assisted laser desorption ionization–time of flight mass spectrometry (MALDI-TOF) (MALDI Biotyper, Bruker). To account for potential polyclonal infection, at least 10 independent colonies identified as H. influenzae or *H. parainfluenzae* were chosen from each plated sample to study their clonal relatedness using PFGE, as previously described (32, 43). One isolate from each PFGE pattern found for each patient episode was selected for further analysis.

### Antimicrobial susceptibility testing.

Antimicrobial susceptibility was assessed by disk diffusion and microdilution using commercial panels (STRHAE2, Sensititre), following the recommended clinical breakpoints of the European Committee on Antimicrobial Susceptibility Testing (EUCAST) and the Clinical and Laboratory Standards Institute (CLSI). Manual microdilution was performed on strains with a MIC for azithromycin <4 mg/L, with testing concentrations ranging from 1 to 512 mg/L.

### Whole-genome sequencing and assembly.

H. influenzae and *H. parainfluenzae* isolates were subjected to WGS, using both short- and long-read sequencing. Genomic DNA was extracted using the QIAamp DNA minikit (Qiagen) and quantified by the QuantiFluor dsDNA System (Promega). For short-read WGS, Nextera XT was used to prepare the libraries, followed by paired-end sequencing on a MiSeq platform (Illumina Inc.). For long-read WGS, Native Barcoding Expansion (EXP-NBD196) and the Ligation Sequencing kit (SQK-LSK109) were used to prepare the libraries, followed by sequencing on FLO-MIN106D flow cells (R9.4.1) (Oxford Nanopore Technologies). A hybrid assembly of short and long reads was produced using the Unicycler pipeline (github.com/rrwick/Unicycler) (44). The assembly statistics were obtained using assembly-stats (github.com/sanger-pathogens/assembly-stats).

### Phylogenetic analysis and antimicrobial resistance determinants.

Phylogenetic analysis was conducted using Snippy (github.com/tseemann/snippy) and RAxML-NG (github.com/amkozlov/raxml-ng) (45), as previously described (46). H. influenzae Hi375 (GenBank accession CP009610) and *H. parainfluenzae* T3T1 (GenBank accession NC_015964) genomes were used as references.

MLST was determined in H. influenzae genomes using the MLST v2.4 software (github.com/tseemann/mlst). *In silico* serotyping was conducted using hicap (github.com/scwatts/hicap) (47), and NTHi were classified into clades based on the presence or absence of 17 accessory genes using patho_typing (github.com/B-UMMI/patho_typing) (48, 49).

Acquired antimicrobial resistance determinants were screened using ResFinder (50). The screening for mutations in genes involved in antibiotic resistance was performed using Geneious R9 (Biomatters), with the H. influenzae Hi375 (CP009610) and *H. parainfluenzae* T3T1 (NC_015964) genomes used as references. Short reads were mapped against 23S rRNA, and the percentage of altered sites was used to estimate the number of 23S rRNA copies with changes.

### Bacterial persistence and genetic adaptation.

Persistent lineages were defined as groups of isolates from the same patient collected over a span of more than 3 months. For H. influenzae, isolates were deemed part of the same lineage if they had the same ST by *in silico* MLST or differed at only one of the MLST loci as SLV. Since they lack a specific MLST schema, *H. parainfluenzae* isolates were grouped into lineages if they differed by three or fewer PFGE bands.

Genome assemblies of persistent strains were annotated using Prokka v1.13.7 (51) with orthologs initially checked in H. influenzae Hi375 (CP009610) or *H. parainfluenzae* T3T1 (NC_015964). To identify the genetic changes accumulated during persistent colonization, assemblies from each persistence episode were aligned using progressiveMauve with Mauve 2.4.0 (52), using the first isolate from each series as the complete genome reference for the lineage. Geneious R9 (Biomatters) was used to search for nonsynonymous SNPs, insertions and deletions (indels), and changes in phase variation and promoter regions. Functional annotation of reference genomes was performed using EggNOG-mapper 2.1.9 (eggnog-mapper.embl.de) (53), and the biological function of the altered genes was assessed using OrthoDB (54).

Identification of genome-integrated prophage sequences used Phaster (55), and those corresponding to intact prophages (score >90) were further considered and named according to Addriaenssens and Brister (56). Phylogeny and classification of prophages were performed with VICTOR (victor.dsmz.de) (57), and prophages found in the genomes of persistent strains were aligned with progressiveMauve.

### Statistical analysis.

Statistical analyses were carried out using the GraphPad Prism 5 software, applying unpaired *t* test or Fisher’s exact test, when appropriate. *P* < 0.05 was considered statistically significant.

### Ethical statement.

This study was in accordance with the Declaration of Helsinki from the World Medical Association. Written informed consent was not required as this was a retrospective and observational study with isolates obtained as part of routine microbiological tests, which was approved by the Clinical Research Ethics Committee of Bellvitge University Hospital (PR252/22). The patient cohort has the approval of the Spanish Health Department for its performance as a postauthorization observational study (cataloged as such by the Spanish Medicines Agency, SPP-AZT-2016-01) and the Clinical Research Ethics Committee of Bellvitge University Hospital (EPA019/16). Patient confidentiality was always protected, and all personal data were anonymized following the current legal normative in Spain (LOPD 15/1999 and RD 1720/2007). Moreover, this project followed Law 14/2007 on Biomedical Research for the management of biological samples in clinical research.

### Data availability.

Sequence reads were deposited in the European Nucleotide Archive (ENA) under the project accession number PRJEB53905. The accession numbers of the reads used in this study are listed in Supplementary Data set S1A and B.
