# Supplementary material for: Genetic Adaptation and Acquisition of Macrolide Resistance in Haemophilus spp. during Persistent Respiratory Tract Colonization in Chronic Obstructive Pulmonary Disease (COPD) Patients Receiving Long-Term Azithromycin Treatment
Source: Microbiol Spectr. 2022 Dec 8;11(1):e03860-22. doi: 10.1128/spectrum.03860-22 (PMC9927455; doi:10.1128/spectrum.03860-22)
Supplement: Supplemental file 3 — Supplemental material. Download spectrum.03860-22-s0003.pdf, PDF file, 3.1 MB [file spectrum.03860-22-s0003.pdf]

Supplementary Table S1. Genetic determinants with genetic changes in three or more cases of *H. influenzae* persistence during azithromycin treatment.

Asterisks indicate that the strain had two genetically altered copies of the gene. ST, sequence type; #SLV, single-locus variant.

Genetic determinant	Locus tag	Accession number	Persistence case				
			**P02-ST139/ST2111#**	**P08-ST107**	**P11-ST2480**	**P11-ST147**	**P13-ST165**
Hemoglobin-haptoglobin binding protein (*hgpB*)	NF38_01815	WP_164927859.1	X*	X	X	X	X
Phosphorylcholine kinase (*licA*)	NF38_07490	WP_005693562.1	X	X	X	X	X
Hemoglobin-haptoglobin binding protein C (*hgpC*)	NF38_01570	WP_010869038.1	X	X	X		X
OmpP1/FadL family transporter (*fadL*)	NF38_03050	WP_038440615.1	X		X	X	X
CMP-Neu5Ac--lipooligosaccharide alpha 2-3 sialyltransferase (*lic3A*)	NF38_03620	WP_038440754.1	X*	X	X		X*
Lipooligosaccharide biosynthesis protein (*lex1*)	NF38_02325	WP_038440349.1	X*	X		X	X
Glycosyltransferase family 2 protein	NF38_07685	WP_038441301.1	X	X	X		X
Glycosyltransferase family 8 protein	NF38_06815	WP_038441170.1	X	X		X	X
Glycosyltransferase family 8 protein	NF38_04110	WP_038441445.1	X	X	X		X
Glycosyltransferase	NF38_06750	WP_005688459.1	X			X	X
Immunoglobulin A1 protease autotransporter precursor	NF38_00020	WP_005693332	X			X	X
Acyltransferase family protein	NF38_03095	WP_038440625.1	X	X			X
Outer membrane protein 2 (*ompP2*)	NF38_04725	WP_005694426	X			X	X
Two-partner secretion (TPS) system translocator (*hmw1B*)	NF38_05835	WP_038441016.1	X*	X*	X*		
Filamentous hemagglutinin N-terminal domain-containing protein (*hmw1A*)	NF38_07770	WP_038441018.1	X*	X*	X		
Transferrin-binding protein-like solute binding protein (*tbpB*)	NF38_09000	WP_038441419.1		X		X	X

Supplementary Table S2. Comparison of GC content and USS/Kb between prophage and genome sequences.
GC: Guanine-cytosine; USS: DNA uptake signal sequences. *Reference strain.

Prophage	Strain	GC content prophage (%)	GC content genome (%)	USS/Kb prophage	USS/Kb genome
Haemophilus phage HUB-P02-ST139-01	HUB-P02-HI01*	39,3	38,1	0,43	0,8
	HUB-P02-HI02	39,3	38,2	0,43	0,8
	HUB-P02-HI19	38,3	38,1	0,54	0,81
Haemophilus phage HUB-P02-ST139-02	HUB-P02-HI01*	40,8	38,1	0,67	0,8
	HUB-P02-HI02	40,8	38,2	0,67	0,8
	HUB-P02-HI19	40,7	38,1	0,69	0,81
Haemophilus phage HUB-P04-HPAR04.05-01	HUB-P04-HP04*	43,9	39,6	0,28	0,71
	HUB-P04-HP07	43,9	39,6	0,28	0,71
	HUB-P04-HP08	43,9	39,6	0,28	0,71
	HUB-P04-HP12	43,9	39,7	0,28	0,71
Haemophilus phage HUB-P08-ST107-01	HUB-P08-HI13	39,8	38,2	0,32	0,79
	HUB-P08-HI14	39,8	38,2	0,32	0,79
	HUB-P08-HI15	39,8	38,2	0,32	0,79
	HUB-P08-HI16	39,8	38,2	0,32	0,79
	HUB-P08-HI17	39,8	38,2	0,32	0,79
	HUB-P08-HI18	39,8	38,2	0,32	0,79
Haemophilus phage HUB-P08-ST107-02	HUB-P08-HI09*	42,3	38,1	0,61	0,8
	HUB-P08-HI10	42,3	38,1	0,61	0,74
Haemophilus phage HUB-P08-ST107-03	HUB-P08-HI13	44,4	38,2	0,44	0,79
	HUB-P08-HI14	44,4	38,2	0,44	0,79
	HUB-P08-HI15	44,4	38,2	0,44	0,79
	HUB-P08-HI16	44,4	38,2	0,44	0,79
	HUB-P08-HI17	44,4	38,2	0,44	0,79
	HUB-P08-HI18	44,4	38,2	0,44	0,79
Haemophilus phage HUB-P11-ST2480-01	HUB-P11-HI01	39,4	38,2	0,41	0,77
	HUB-P11-HI02*	39,4	38,2	0,41	0,77
	HUB-P11-HI03	39,4	38,2	0,41	0,77
	HUB-P11-HI04	39,4	38,2	0,41	0,77
	HUB-P11-HI05	39,5	38,2	0,41	0,77
	HUB-P11-HI06	39,5	38,2	0,41	0,77
	HUB-P11-HI07	39,5	38,2	0,41	0,77
Haemophilus phage HUB-P11-ST2480-02	HUB-P11-HI01	39,5	38,2	0,26	0,77
	HUB-P11-HI02*	39,5	38,2	0,26	0,77
	HUB-P11-HI03	39,5	38,2	0,26	0,77
	HUB-P11-HI04	39,5	38,2	0,26	0,77
	HUB-P11-HI05	39,5	38,2	0,26	0,77
	HUB-P11-HI06	39,5	38,2	0,26	0,77
	HUB-P11-HI07	39,5	38,2	0,26	0,77
Haemophilus phage HUB-P11-ST147-01	HUB-P11-HI09*	40,4	38,1	0,6	0,78
	HUB-P11-HI011	40,4	38,1	0,6	0,78

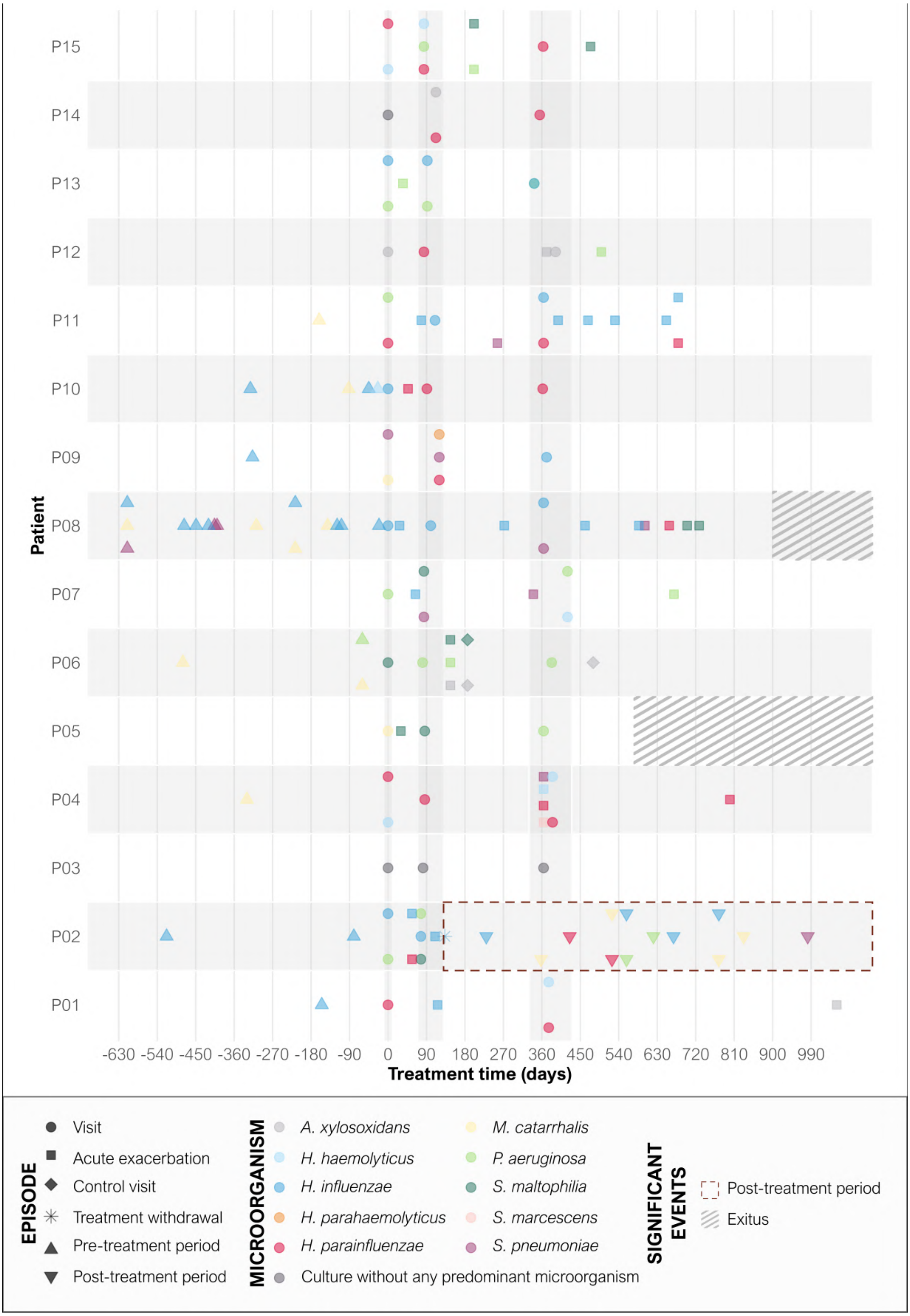

Supplementary Figure S1. Timeline illustrating the microorganisms isolated from respiratory samples in the severe COPD patients with long-term azithromycin therapy included in this study. The vertical shaded regions represent the time intervals between programmed visits: prior to start azithromycin therapy (V1), three (V2), and twelve (V3) months after starting therapy.

A) Haemoglobin-haptoglobin binding protein B (*hgpB*)

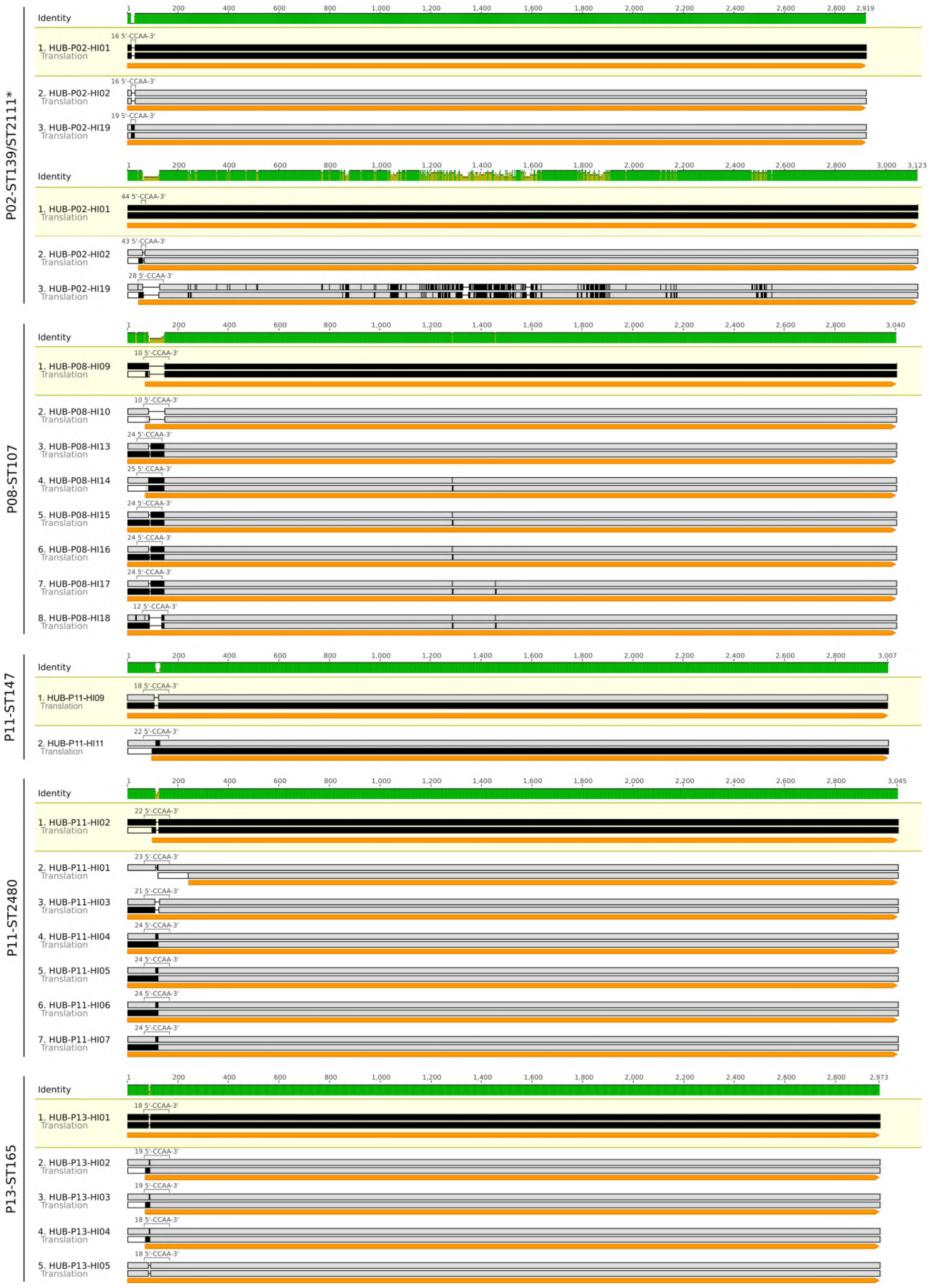

Supplementary Figure S2 (continued to next page).

B) Phosphorylcholine kinase LicA (*licA*)

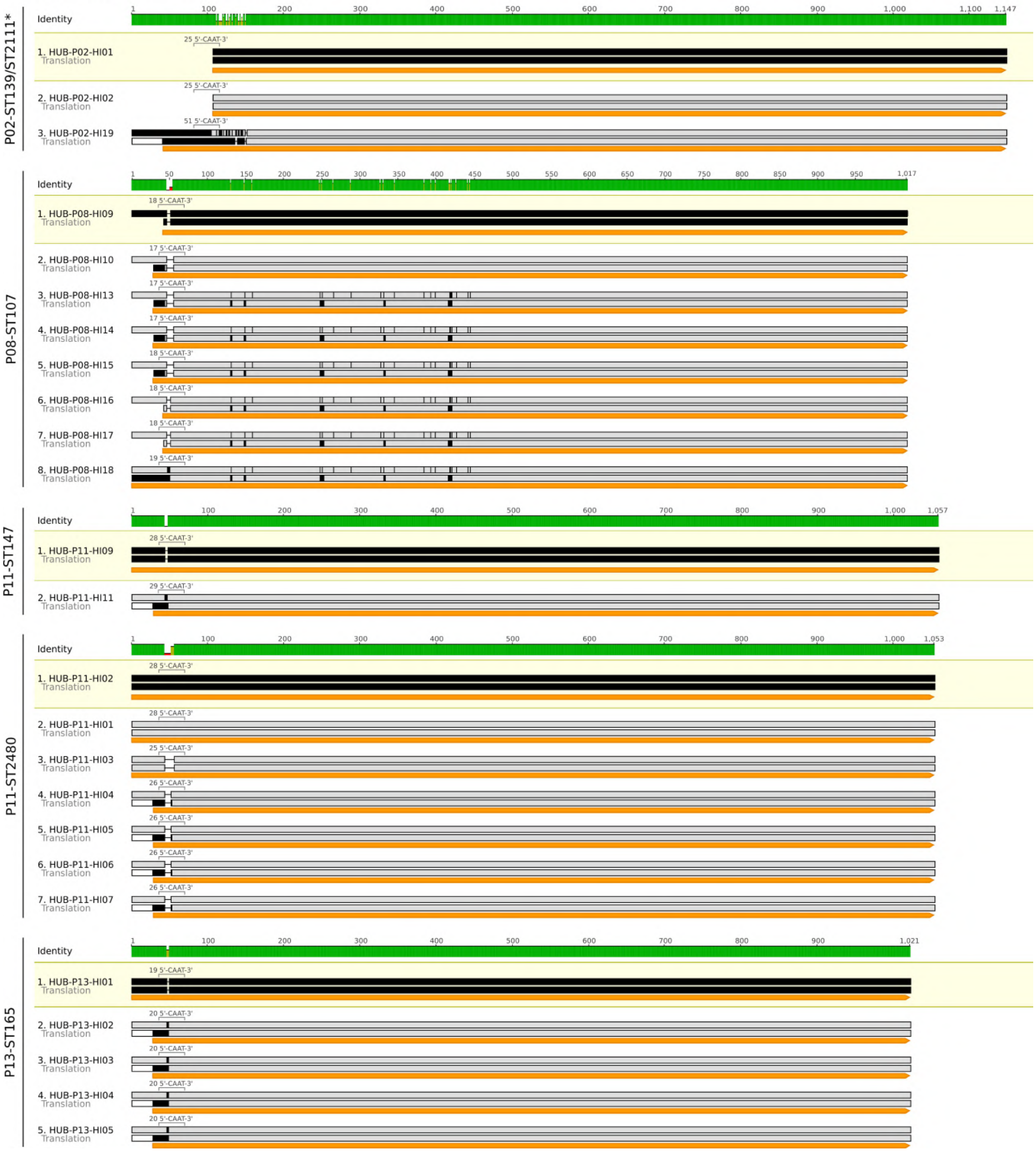

Supplementary Figure S2 (continued to next page).

C) Haemoglobin-haptoglobin binding protein C (*hgpC*)

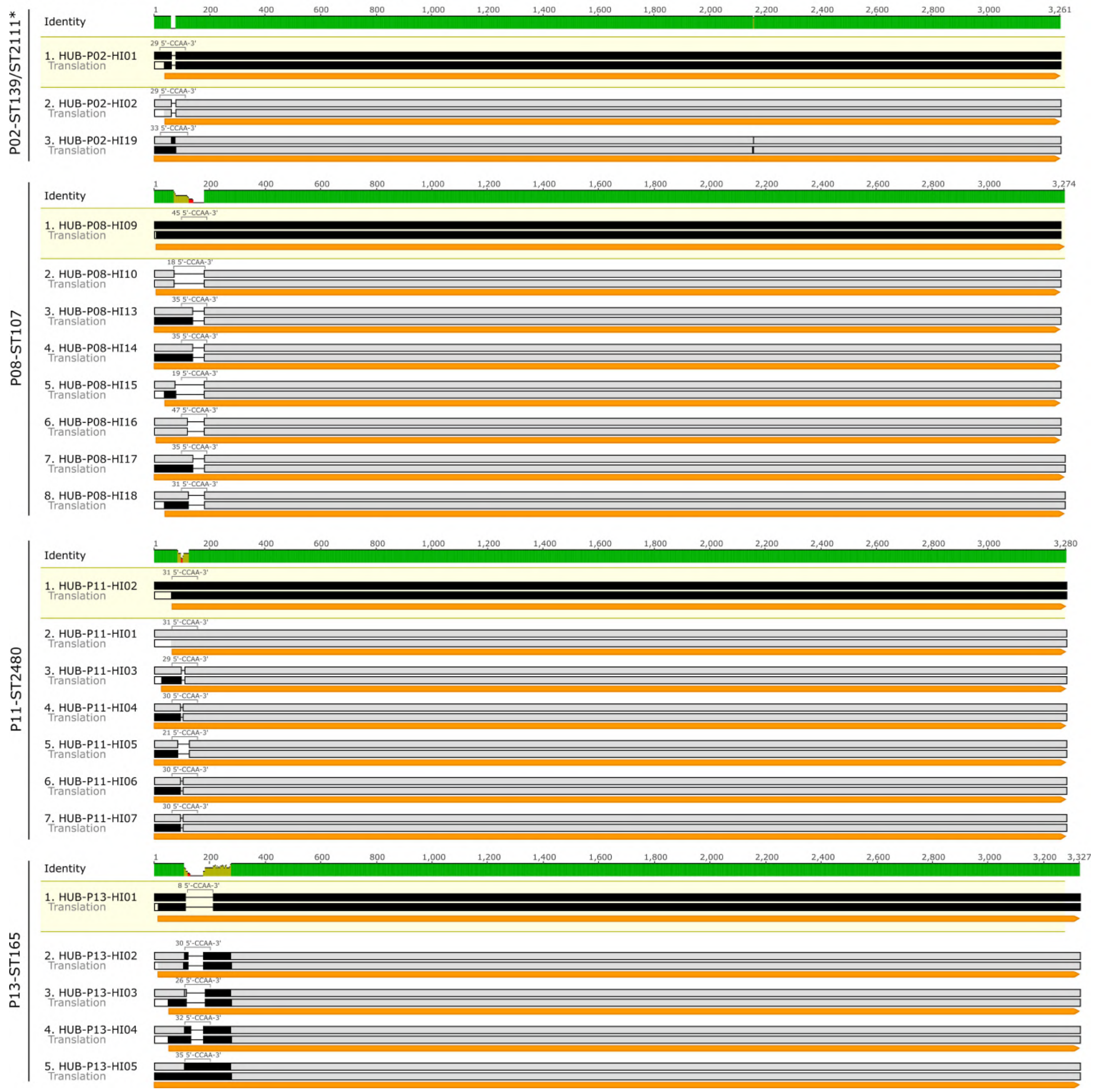

Supplementary Figure S2 (continued to next page).

D) OmpP1/FadL family transporter (*fadL*)

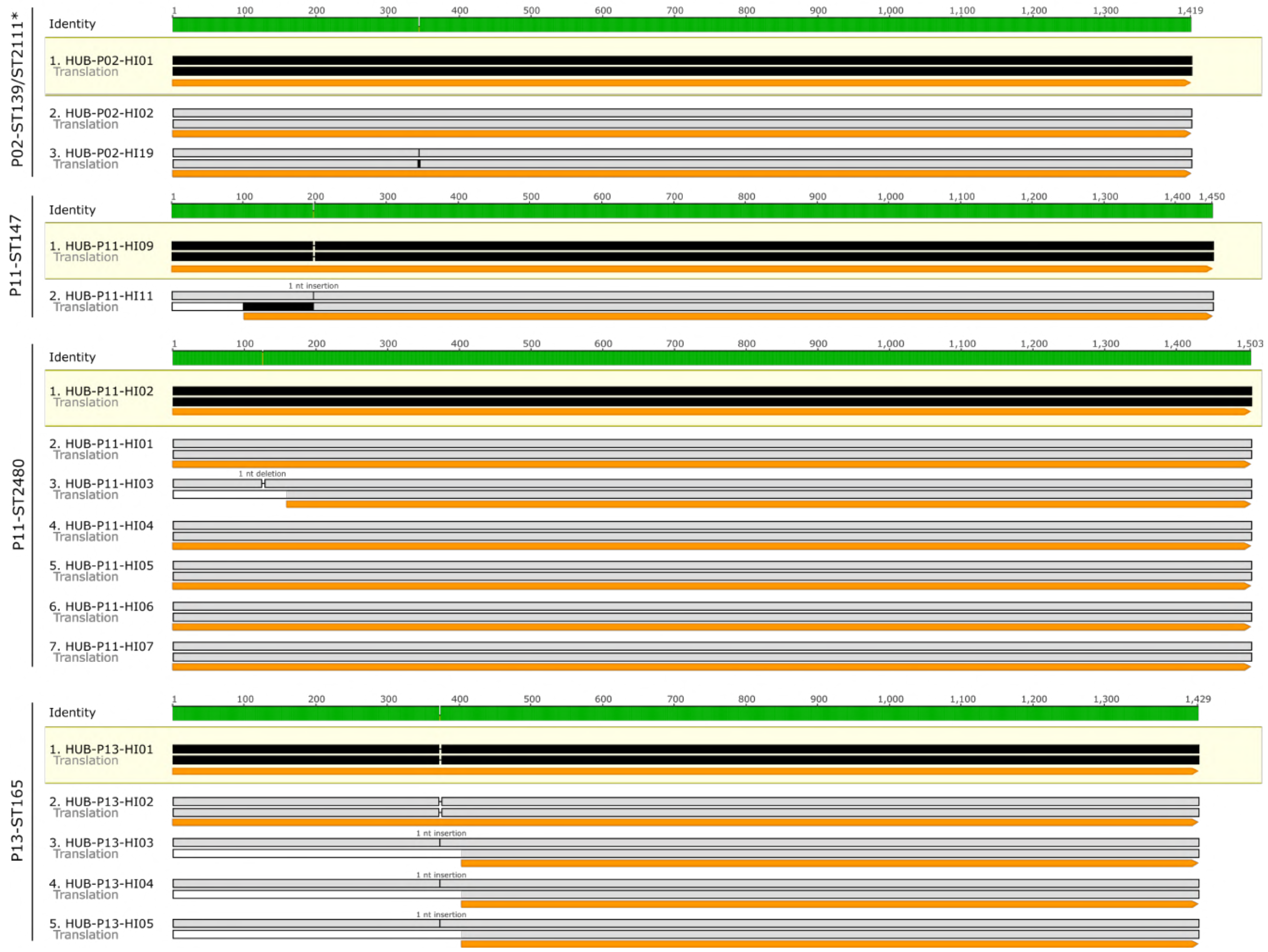

Supplementary Figure S2 (continued to next page).

E) CMP-Neu5Ac--lipooligosaccharide alpha 2-3 sialyltransferase (*lic3A*)

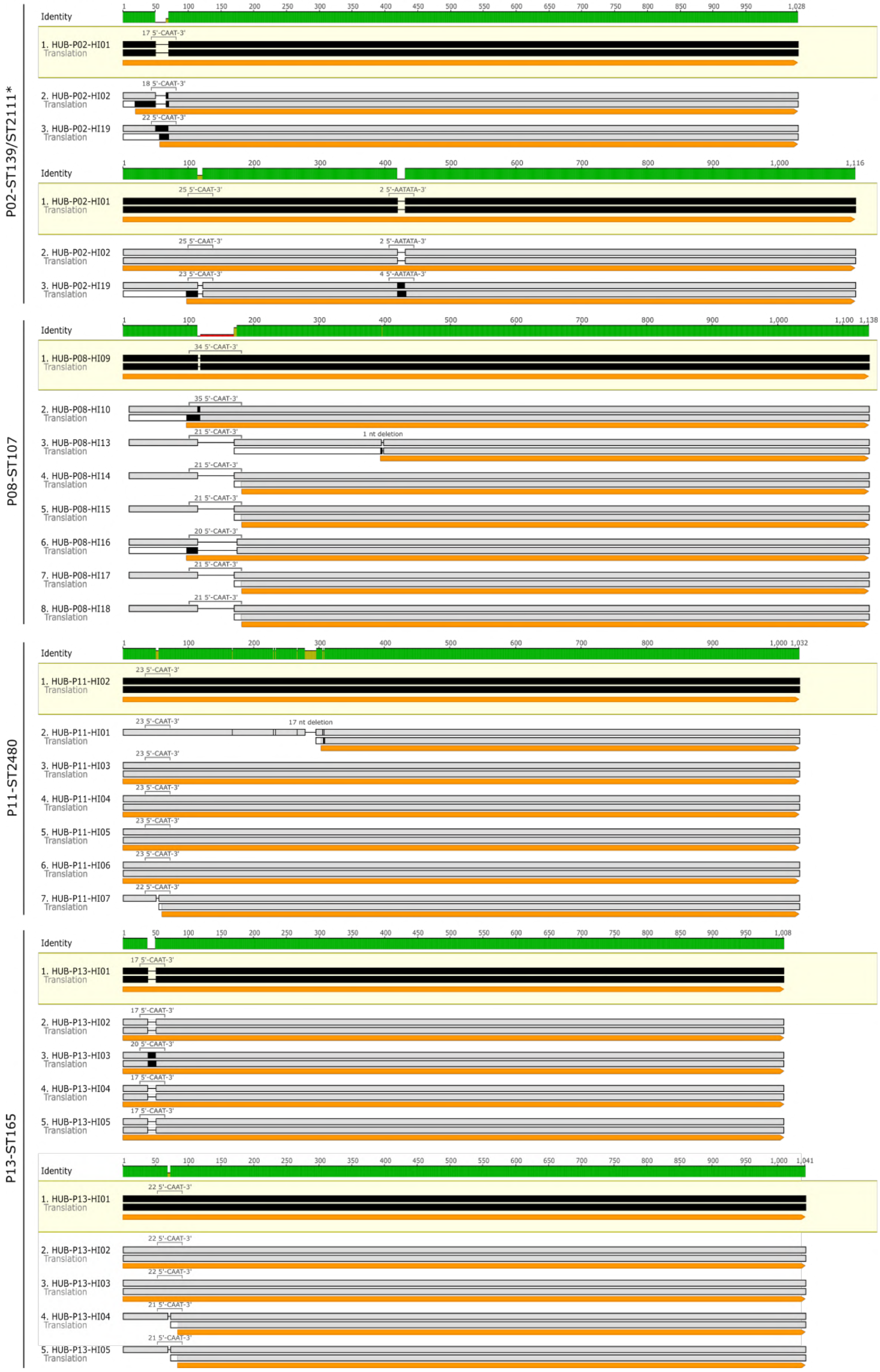

Supplementary Figure S2 (continued to next page).

F) Lipooligosaccharide biosynthesis protein Lex-1(*lex1*)

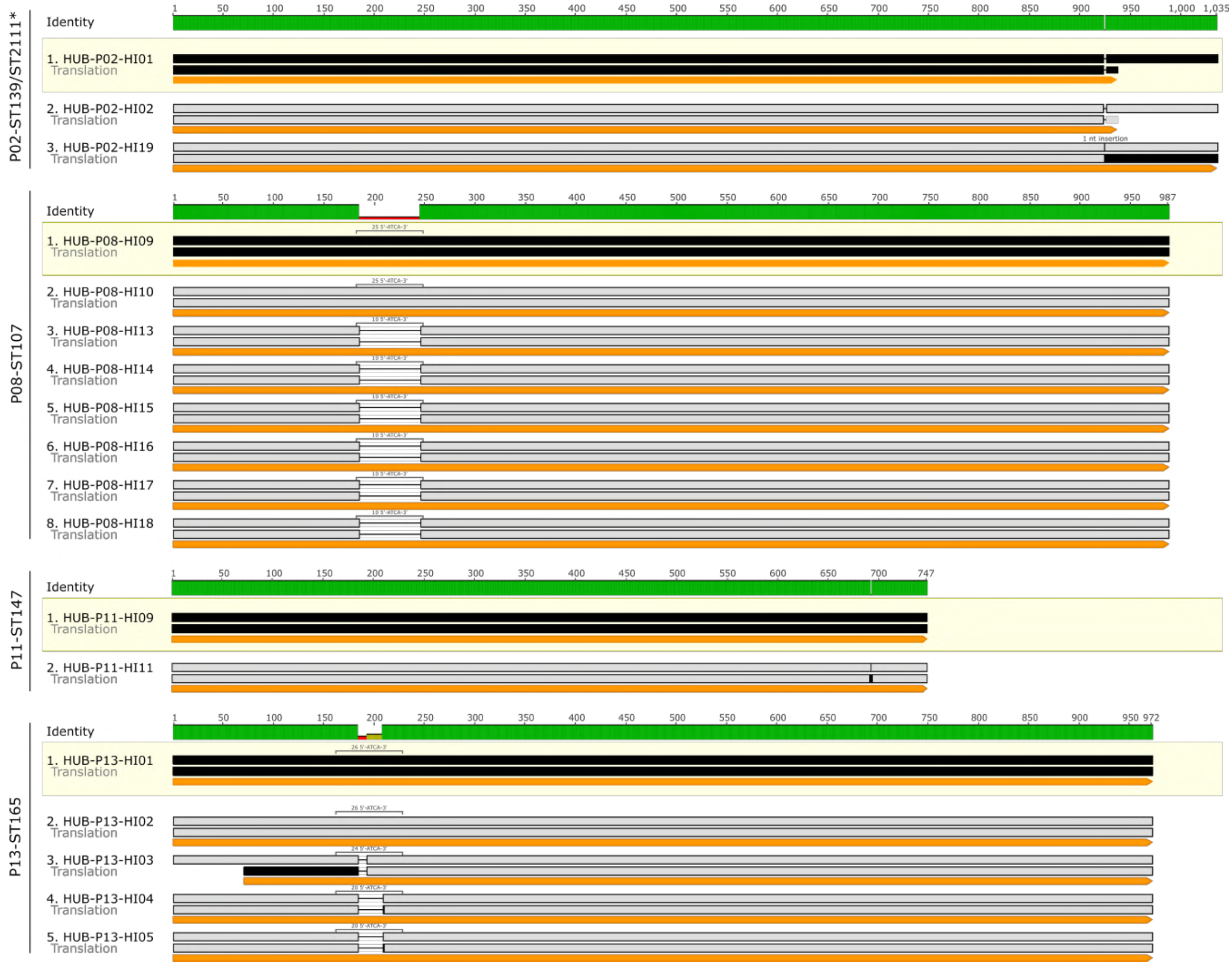

Supplementary Figure S2 (continued to next page).

G) Glycosyltransferase family 2 protein (NF38_07685)

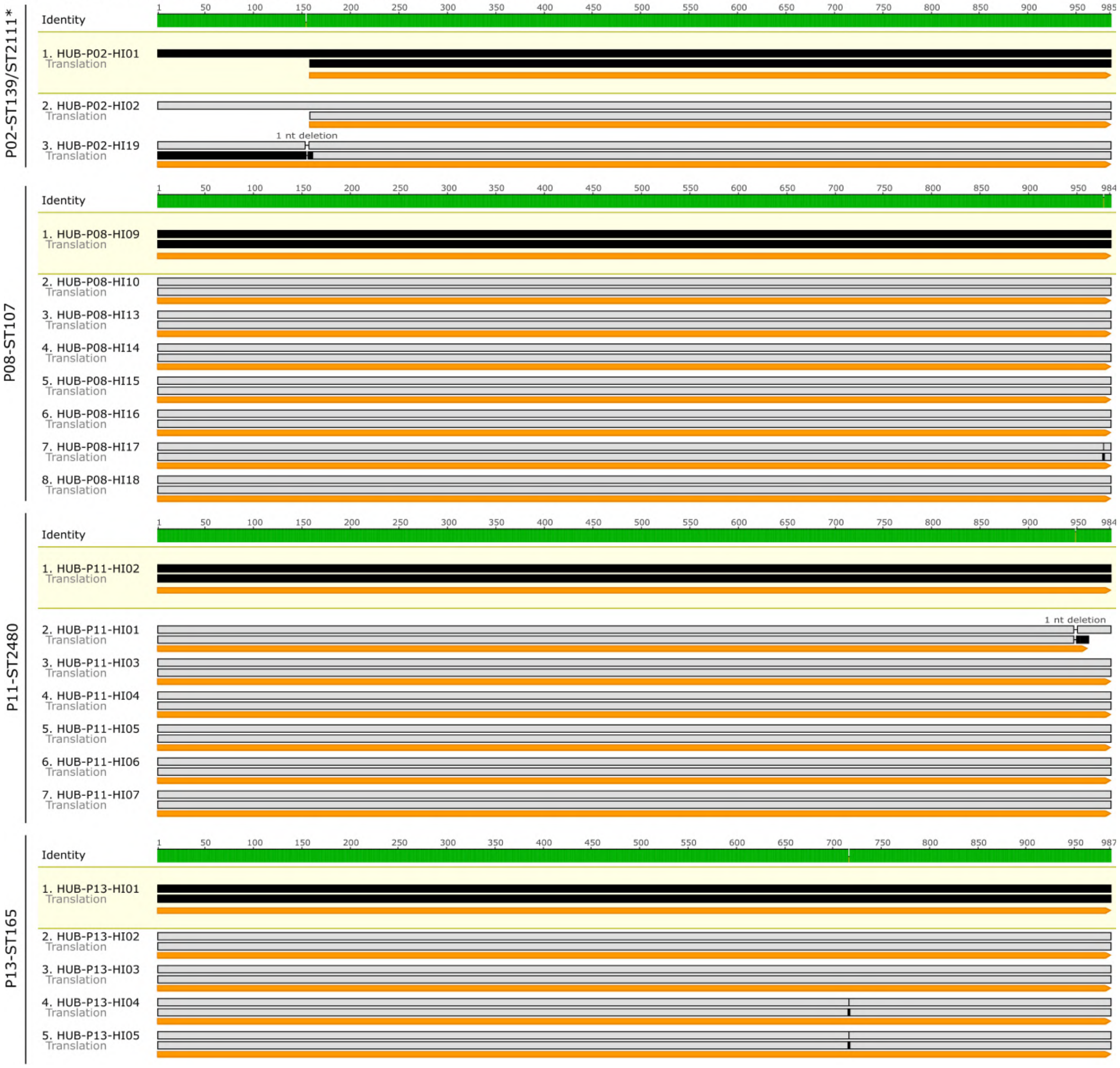

Supplementary Figure S2 (continued to next page).

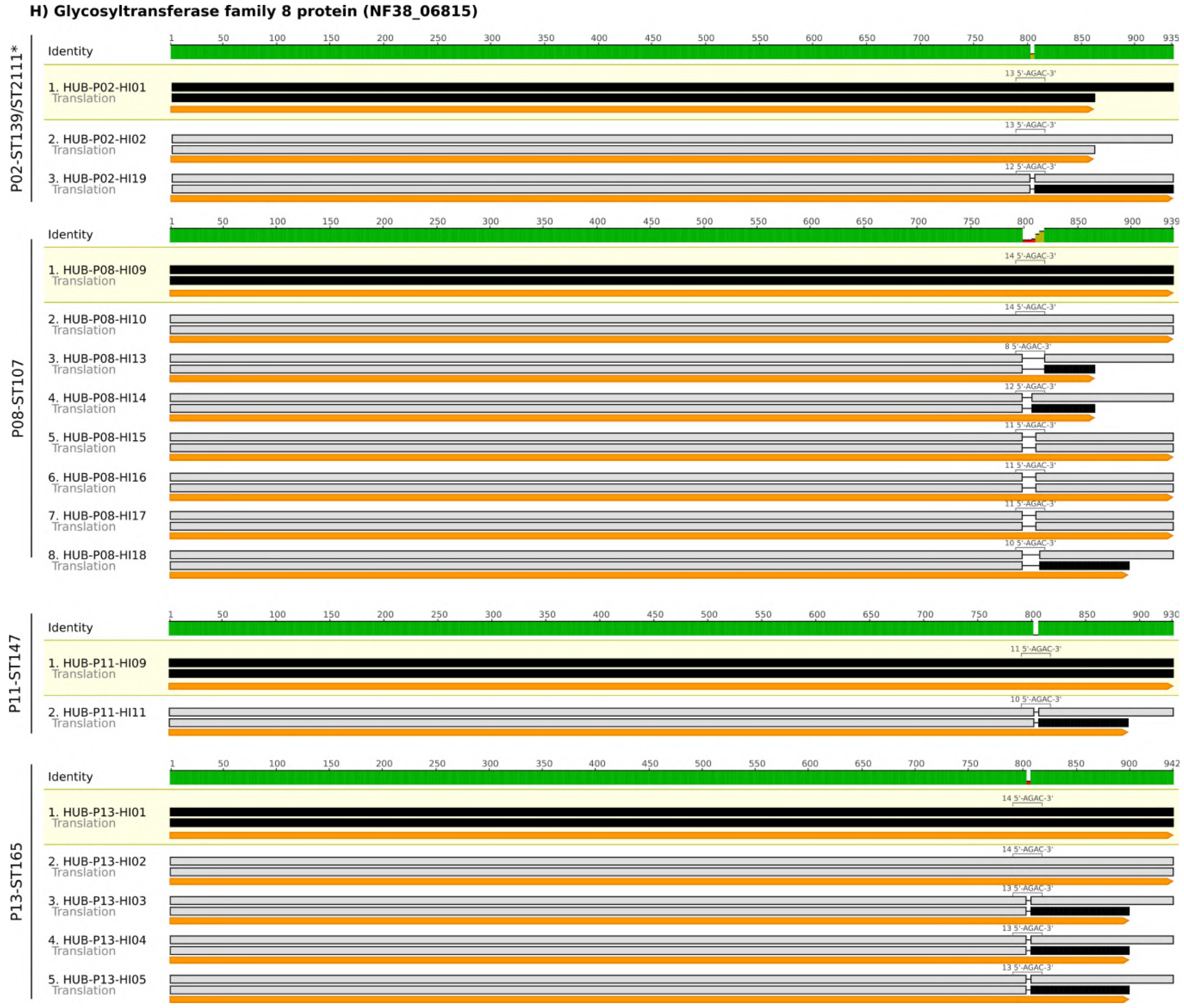

Supplementary Figure S2 (continued to next page).

I) Glycosyltransferase family 8 protein (NF38_04110)

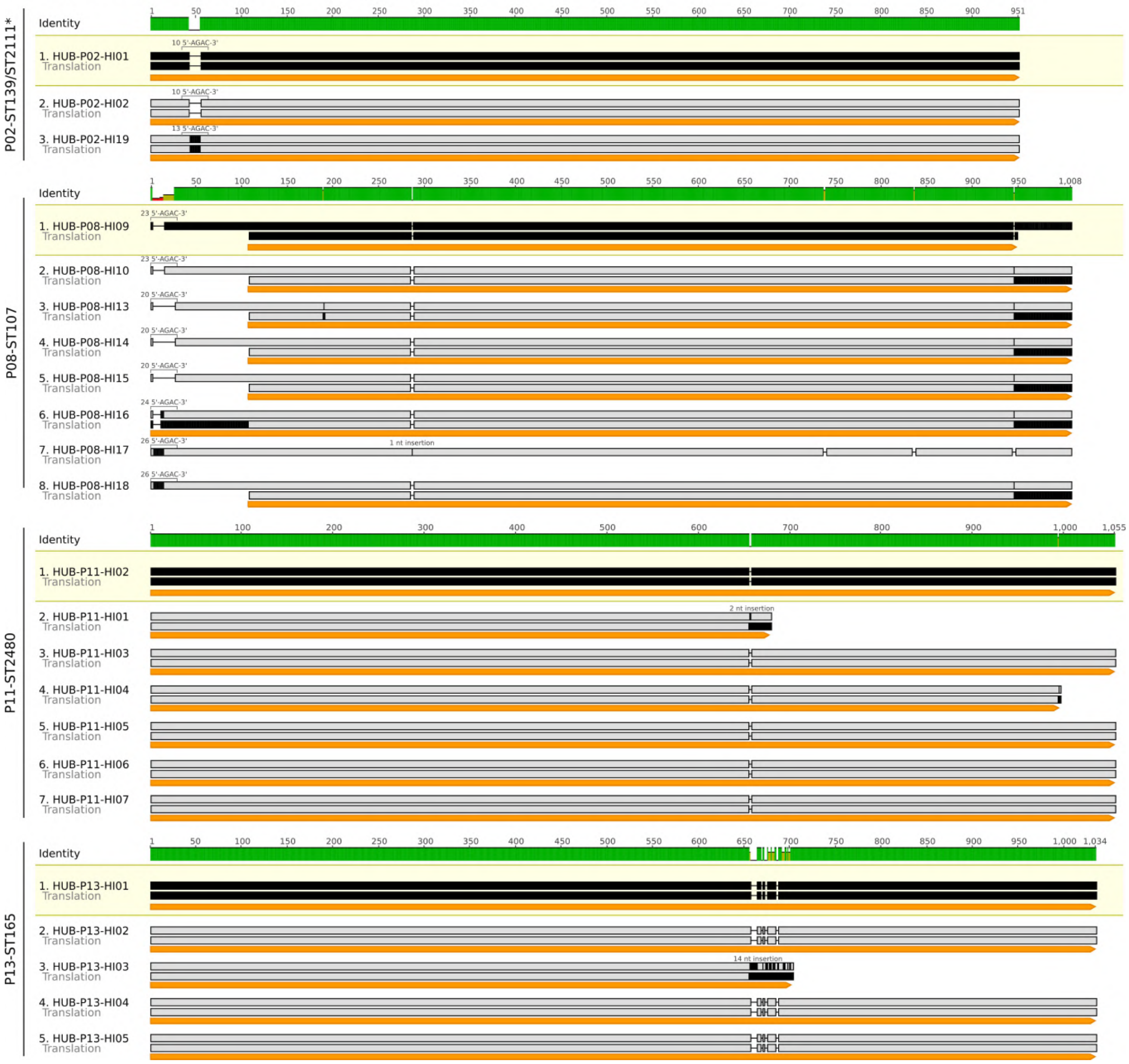

Supplementary Figure S2. Alignment of genes that showed genetic changes in four or more cases of *H. influenzae* persistence. (A) *hgpB*, coding by hemoglobin-haptoglobin binding protein B; **(B)** *licA*, coding by phosphorylcholine kinase LicA; **(C)** *hgpC*, coding by hemoglobin-haptoglobin binding protein C; **(D)** *fadL* (*ompP1*), coding by an outer membrane transporter; **(E)** *lic3A*, coding by CMP-Neu5Ac--lipooligosaccharide alpha 2-3 sialyltransferase; **(F)** *lex1*, coding for lipooligosaccharide biosynthesis protein Lex-1; and **(G-I)** genes coding by glycosyltransferase family 2 and 8 proteins. The reference strains of each persistence case are highlighted in yellow. The numbers above the identity bar show the gene position (nt). The aligned gene, its translation and potential open reading frames (orange arrows) are shown for each strain. Changes from the reference strain are indicated in black. *SLV, single-locus variant.

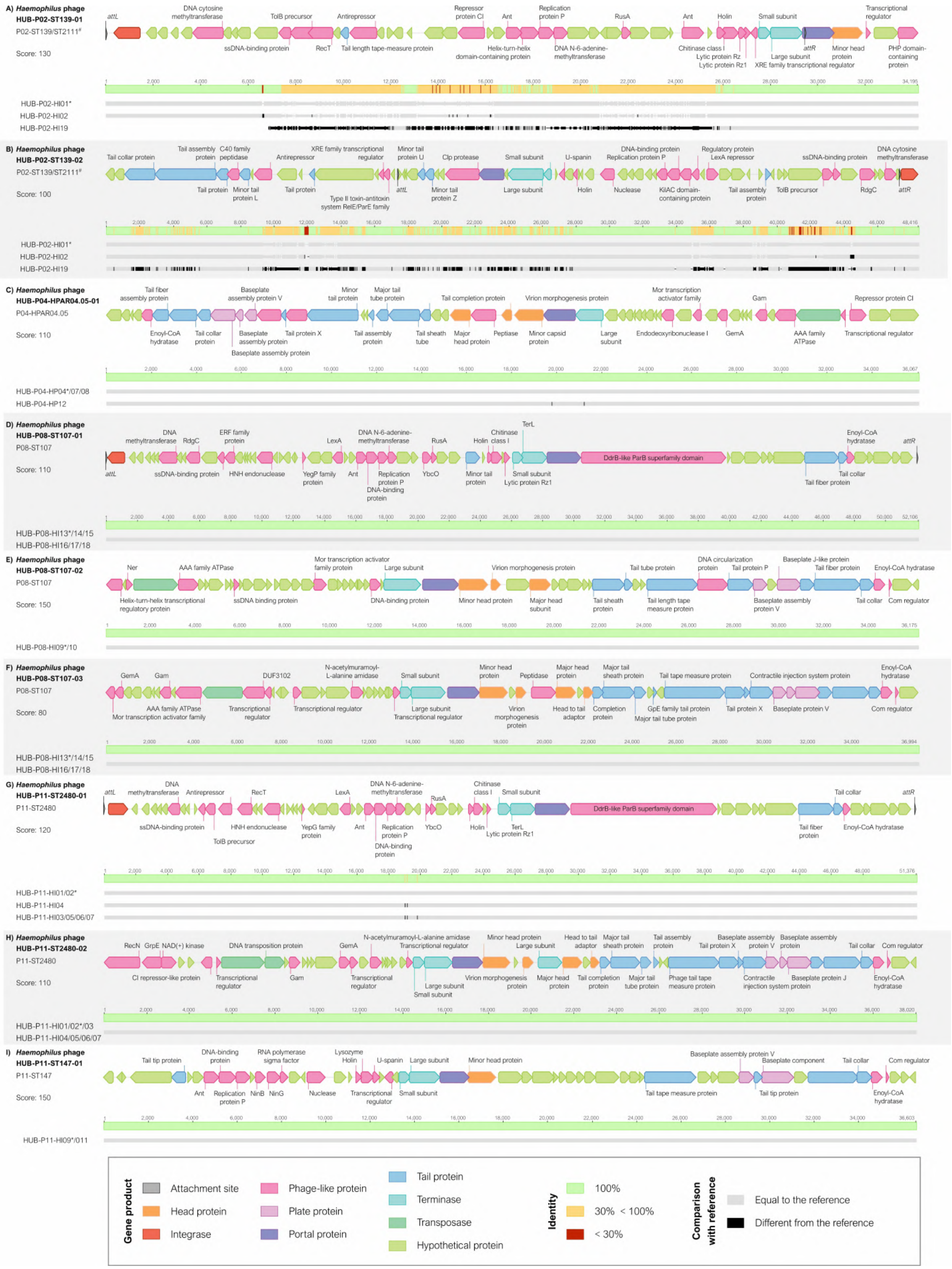
A) Haemophilus phage HUB-P02-ST139-01
P02-ST139/ST2111#
Score: 130
HUB-P02-HI01*
HUB-P02-HI02
HUB-P02-HI19
B) Haemophilus phage HUB-P02-ST139-02
P02-ST139/ST2111#
Score: 100
HUB-P02-HI01*
HUB-P02-HI02
HUB-P02-HI19
C) Haemophilus phage HUB-P04-HPAR04.05-01
P04-HPAR04.05
Score: 110
HUB-P04-HP04*/07/08
HUB-P04-HP12
D) Haemophilus phage HUB-P08-ST107-01
P08-ST107
Score: 110
DdrB-like ParB superfamily domain
HUB-P08-HI13*/14/15
HUB-P08-HI16/17/18
E) Haemophilus phage HUB-P08-ST107-02
P08-ST107
Score: 150
HUB-P08-HI09*/10
F) Haemophilus phage HUB-P08-ST107-03
P08-ST107
Score: 80
HUB-P08-HI13*/14/15
HUB-P08-HI16/17/18
G) Haemophilus phage HUB-P11-ST2480-01
P11-ST2480
Score: 120
DdrB-like ParB superfamily domain
HUB-P11-HI01/02*
HUB-P11-HI04
HUB-P11-HI03/05/06/07
H) Haemophilus phage HUB-P11-ST2480-02
P11-ST2480
Score: 110
HUB-P11-HI01/02*/03
HUB-P11-HI04/05/06/07
I) Haemophilus phage HUB-P11-ST147-01
P11-ST147
Score: 150
HUB-P11-HI09*/011
Gene product
Attachment site
Head protein
Integrase
Phage-like protein
Plate protein
Portal protein
Tail protein
Terminase
Transposase
Hypothetical protein
Identity
100%
30% < 100%
< 30%
Comparison with reference
Equal to the reference
Different from the reference

Supplementary Figure S3. Schematic representation of genome-integrated prophages detected in persistent *Haemophilus* spp. Intact prophages detected by Phaster (score ≥ 90) were included. *Haemophilus* phage HUB-P08- ST107-03 was included despite having a score <90 because strains HUB-P08-HI13 to HUB-P08-HI18 showed a 36 kb insertion compared to the reference strain (HUB-P08-HI09). The direction of the arrows indicates the direction of the reading frame of the genes. The first prophage found in a closed genome over time in each case was used as a reference (*), and the percentage of identity of each sequence with respect to the reference is indicated to the right of each horizontal grey line. **(A) *Haemophilus* phage HUB-P02-ST139-01**. In HUB-P02-HI19 had a deletion of the downstream region, including genes coding for integrase, DNA cytosine methyltransferase, and 8 hypothetical proteins. This was followed by a region of high genetic variability, affecting 29 genes with respect to the reference strain (HUB-P02-HI01). **(B) *Haemophilus* phage HUB-P02-ST139-02**. This prophage accumulated 260 SNPs and the last isolated strain (HUB-P02-HI19) showed 90.4% of identity to the reference strain (HUB-P02-HI01). The location of this prophage within the genome is the same over time, suggesting the probable recombination with another related prophage from an unknown donor. **(C) *Haemophilus* phage HUB-P04-HPAR 04.05-01**. Strain HUB-P04-HP10 lost 115,160 bp corresponding to the prophage and its surrounding regions (37,453 bp downstream and 41,640 bp upstream). The prophage genomes from serial isolates were nearly identical. **(D) *Haemophilus* phage HUB-P08- ST107-01**. The last six strains of the ST107 lineage (HUB-P08-HI13 to HUB-P08-HI18) acquired this prophage, inserting 52.2 kb into these genomes in a gene for tRNA-Leu. **(E) *Haemophilus* phage HUB-P08-ST107-02**. This prophage was only detected in the first two isolates of the lineage (HUB-P08-HI09 and HUB-P08-HI10) and was absent from the later six strains, as seen by a 36.2 kb deletion in the genome alignment (Figure 5). **(F) *Haemophilus* phage HUB-P08-ST107-03**. The last six strains of the ST107 lineage (HUB-P08-HI13 to HUB-P08-HI18) acquired this prophage, inserting 37.0 kb into these genomes in an intragenic non-coding region between genes encoding homoserine O-acetyltransferase and YcaO-like proteins. **(G) *Haemophilus* phage HUB-P11-ST 2480-01**. **(H) *Haemophilus* phage HUB-P11-ST2480-02**. **(I) *Haemophilus* phage HUB-P11-ST147-01**. #SLV, single-locus variant.

Supplementary Dataset S3. Biological functions of genes from reference genomes. Functional annotation was based on orthology assignments using 70% percentage identity, 60% minimum of query coverage, and 60% minimum of subject coverage in EggNOG-mapper 2.1.9. Each gene may be annotated with more than one biological function, so that the sum of all the annotations may be greater than the total number of genes. * Total number of genes annotated by Prokka v1.13.7.

Biological function	HUB-P02-HI01	HUB-P08-HI09	HUB-P11-HI02	HUB-P11-HI09	HUB-P13-HI01	HUB-P04-HP04	HUB-P11-HP02
**Total genes***	1766	1725	1879	1818	1756	1841	1836
**Amino acid transport and metabolism**	134	133	132	131	136	136	136
**Carbohydrate transport and metabolism**	89	87	80	86	88	92	78
**Cell cycle control, cell division, chromosome partitioning**	32	33	33	33	34	30	31
**Cell motility**	7	9	7	9	12	7	9
**Cell wall/membrane/envelope biogenesis**	121	122	118	121	126	125	121
**Coenzyme transport and metabolism**	91	91	92	91	91	95	102
**Defense mechanisms**	18	20	17	18	23	19	18
**Energy production and conversion**	108	110	109	109	107	126	124
**Extracellular structures**	0	0	0	0	0	1	2
**Inorganic ion transport and metabolism**	111	116	111	113	122	171	147
**Intracellular trafficking, secretion, and vesicular transport**	55	52	51	56	56	48	54
**Lipid transport and metabolism**	36	37	40	41	36	42	38
**Nucleotide transport and metabolism**	96	97	95	95	97	94	94
**Post-translational modification, protein turnover, and chaperones**	72	71	70	72	75	77	83
**Replication, recombination and repair**	95	93	117	104	99	92	119
**RNA processing and modification**	1	1	1	1	1	1	1
**Secondary metabolites biosynthesis, transport, and catabolism**	16	16	15	15	16	17	16
**Signal transduction mechanisms**	25	25	25	26	27	25	26
**Transcription**	94	92	93	101	92	89	90
**Translation, ribosomal structure and biogenesis**	178	179	178	181	180	186	187
**Unknown**	466	421	572	469	423	472	463

Supplementary Dataset S4. Surrounding regions of *Haemophilus* phage HUB-P04-HPAR04.05-01 which were lost in HUB-P04-HP10.

Product	Upstream/Downstream	Length (bp)
Bifunctional peptide-methionine (S)-S-oxide reductase MsrA/peptide-methionine (R)-S-oxide reductase MsrB	Downstream	1,062
Carboxymuconolactone decarboxylase family protein	Downstream	546
DMT family transporter	Downstream	867
DMT family transporter	Downstream	477
EmrA/EmrK family multidrug efflux transporter periplasmic adaptor subunit	Downstream	1,17
Excinuclease ABC subunit UvrA	Downstream	2,832
Fe(3+) dicitrate ABC transporter ATP-binding protein FecE	Downstream	768
Fe(3+) dicitrate ABC transporter permease subunit FecD	Downstream	984
Fe(3+)-dicitrate ABC transporter substrate-binding protein FecB	Downstream	891
Formate/nitrite transporter family protein	Downstream	795
FRG domain-containing protein	Downstream	1,02
Hypothetical protein	Downstream	2,418
Hypothetical protein	Downstream	372
Hypothetical protein	Downstream	336
Hypothetical protein	Downstream	312
Hypothetical protein	Downstream	1,296
Hypothetical protein	Downstream	1,074
Hypothetical protein	Downstream	5,295
Iron-dicitrate ABC transporter permease FecC	Downstream	990
LysR family transcriptional regulator	Downstream	891
MFS transporter	Downstream	1,527
NAD(P)H-dependent oxidoreductase	Downstream	579
pgaB 1	Downstream	1,164
PTS mannose transporter subunit IIAB	Downstream	969
PTS mannose transporter subunit IID	Downstream	834
PTS mannose/fructose/sorbose transporter subunitIIC	Downstream	798
Redoxin family protein	Downstream	477
rhodanese-like domain-containing protein	Downstream	369
Single-stranded DNA-binding protein	Downstream	468
YdbH family protein	Downstream	2,736
YnbE family lipoprotein	Downstream	207
3'(2'),5'-bisphosphate nucleotidase CysQ	Upstream	807
5'/3'-nucleotidase SurE	Upstream	741
6-phosphogluconolactonase	Upstream	699
Acid phosphatase AphA	Upstream	708
ADP compounds hydrolase NudE	Upstream	546
ADP-glyceromanno-heptose 6-epimerase	Upstream	927
Alpha/beta hydrolase	Upstream	867
ATP-dependent protease subunit HslV	Upstream	531
DedA family protein	Upstream	576
Deoxyribose-phosphate aldolase	Upstream	675
DMT family transporter	Upstream	906
DNA recombination protein RmuC	Upstream	1,611
Galactose/methyl galactoside ABC transporter ATP-binding protein MglA	Upstream	1,512
Galactose/methyl galactoside ABC transporter permease MglC	Upstream	1,128

Glucose-6-phosphate dehydrogenase	Upstream	1,482
GNAT family N-acetyltransferase	Upstream	393
HslU--HslV peptidase ATPase subunit	Upstream	1,335
Hsp33 family molecular chaperone HslO	Upstream	867
Hypothetical protein	Upstream	189
Hypothetical protein	Upstream	939
Hypothetical protein	Upstream	333
Hypothetical protein	Upstream	417
Hypothetical protein	Upstream	276
Lipopolysaccharide heptosyltransferase II	Upstream	1,044
Lipopolysaccharide heptosyltransferase RfaC	Upstream	954
Murein hydrolase activator NlpD	Upstream	1,233
Oligopeptide transporter, OPT family	Upstream	2,001
Peptidylprolyl isomerase	Upstream	639
Phosphoenolpyruvate carboxykinase (ATP)	Upstream	1,617
Porin	Upstream	1,08
Proline--tRNA ligase	Upstream	1,716
Ribonuclease E inhibitor RraB	Upstream	429
Ribosome-associated heat shock protein Hsp15	Upstream	396
SIMPL domain-containing protein	Upstream	714
Substrate-binding domain-containing protein	Upstream	999
TIGR00645 family protein	Upstream	558
tRNA pseudouridine(13) synthase TruD	Upstream	1,023
xylA	Upstream	1,32
xylB	Upstream	1,482
xylR	Upstream	1,164
